# Mapping research on the social impact of the arts: what characterises the field?

**DOI:** 10.12688/openreseurope.14147.1

**Published:** 2021-10-18

**Authors:** Sofia Lindström Sol, Cia Gustrén, Gustaf Nelhans, Johan Eklund, Jenny Johannisson, Roger Blomgren

**Affiliations:** 1 The Swedish School of Library and Information Science (SSLIS), University of Borås, Borås, Sweden

**Keywords:** artistic intervention, effects of culture, bibliometric analysis, cultural policy, arts and culture

## Abstract

This article explores the broad and undefined research field of “the social impact of the arts”. The effects of art and culture are often used as justification for public funding, but the research on these interventions and their effects is unclear. Using a co-word analysis of over 10,000 articles published between 1990 and 2020, we examined the characteristics of the field as we have operationalised it through our searches. Since 2015, the research field of “the social impact of art” has expanded and consists of different epistemologies and methodologies, summarised in largely overlapping subfields belonging to the social sciences/humanities, arts education, and arts and health/therapy. In formal or informal learning settings, studies of theatre/drama as an intervention to enhance skills, well-being, or knowledge among children are most common. A study of the research front, operationalised as the bibliographic coupling of the most cited articles in the data set, confirmed the co-word analysis and revealed new themes that together form the ground for insight into research on the social impact of the arts. As such, this article can inform discussions on the social value of the arts and culture.

## Introduction

The notion that art impacts society beyond its aesthetic value has a long history, dating back to Aristotle’s concept of catharsis ( Belfiore & Bennett, 2007). Since the 1990s, the social effects of the arts have increasingly been explored by researchers, policymakers, and other stakeholders ( Belfiore, 2002; Belfiore & Bennett, 2010; Gray, 2007), and “social impact” often forms a justification for public funding of the arts ( Belfiore, 2002; Gray, 2007). For example, in 2016–2018, the Swedish government funded arts projects in socio-economically marginalised areas to advance democratic ideals and cultural participation, inspired by the Art Council England’s Creative Spaces and People.

This study is written in the context of AMASS (Acting on the Margins: Arts as Social Sculpture), a Horizon 2020 EU-funded project aiming to address aspects of marginalisation in Europe through arts and creative activities ( AMASS, 2019). The testbeds, comprising of various art projects, are situated in Malta, Finland, Hungary, Czech Republic, Italy, and Portugal, countries with a marginal position, geographically and culturally, in the EU. As an overarching project, the AMASS is part of a policy trend to explore the potential of the arts to address social marginalisation in a changing Europe.

Thus, from a policy and practitioners’ perspective, the arts are often laden with positive value, although some research argues that the arts exist only for those who can decipher them: a real impact of the arts can exist only for a small elite of educated people ( Bourdieu, 1984). The mechanisms of art’s impact (either in the form of consumption or production of the arts) on individuals and societies are still unclear ( Belfiore, 2002; Gilmore, 2014), and the research field of “the social impact of the arts” is vaguely defined and difficult to overlook.

This article aims to characterise the field through an overview of trends in studies on the social impact of the arts as a broadly defined research area. The aim is driven by conceptual ambition. What, when delineated and defined, are the major themes found in this research field? What are the characteristics of the ‘research front’ in the social impact of the arts?

If policy on arts and culture is to achieve social impact, the thematic variability and potential theoretical and empirical insights need to be mapped. Through this study, we wish to inform debates on the social effects of the arts and provide cultural policy scholars and artist-researchers with insights into categories, patterns, and trends in the research field of the social impact of the arts. In a previous study ( Gustrén *et al.*, 2021), we have analysed ‘grey literature’ on the social impact of the arts. In this study, we focus on peer-reviewed research in English, a selection discussed below.

### Surveying a field: a literature review

When surveying a clearly defined field of research, scholars commonly use the systematic literature review (SLR) to map and assess current knowledge ( Kitchenham, 2004; O’Brien & McGuckin, 2019). In the field of the art’s social impact, studies such as Daykin *et al.* (2008); Jindal-Snape *et al.* (2018) and Young *et al.* (2016) give us valuable insight into topics such as arts’ effects on adolescent health and behaviour, children’s general academic performance, and on cognitive functioning in elderly individuals with dementia. This study takes a broader grasp of an unclearly defined field. We do not perform a strict SLR but explore a more open approach where a vital objective is *to illustrate the potential variety in the totality of different understandings of art’s social impact displayed in the research literature*. However, in line with the SLR method, we attempted to analyse available research as thoroughly, fairly, and with as little bias as possible ( Kitchenham, 2004; O’Brien & McGuckin, 2019).

In the following section, we will outline the analysis, performed in two general steps. The first step aimed to delineate the field. We formulated a review protocol to collect data, which we thematised and visualised through co-word analysis. To identify the characteristics of the emerged themes, we performed a qualitative analysis by reading selected articles. In the second step of the analysis, we performed a bibliographic coupling on the reference lists of the documents in the set to identify the research front, consisting of articles of similar thematic content, defined as the most cited articles in the data. This generated a new set of themes which were also qualitatively assessed through a close reading of selected, highly cited articles within the set. The article is concluded by discussing the implications and limitations of our findings and making suggestions for further research.

## Methods

### Delineating the field

Firstly, we conducted a pilot study that aimed to identify relevant databases and search strings. The pilot study included two university librarians who conducted literature searches in Web of Science, SCOPUS, Art & Architecture Source (EBSCO), and general search engines such as Google, applying a broad set of keywords.

Based on the pilot study results, we concluded that the review should include additional databases over scholarly publications but exclude general search engines since they provided irrelevant hits. The keywords should be limited to generate more relevant hits in databases. For the study, the delimitation of the search was motivated in line with the AMASS project, thus defining arts as *mainly* performing and visual arts. Secondly, we defined “social impact” as relating to issues of marginalisation targeted by the project, such as poverty, minority issues, and refugees, i.e., in terms of underserved or marginalised communities, populations, or individuals. We also limited our searches to matters relating to effects that addresses their situation.

In the ambition to generate more relevant hits in databases, we limited the keywords within the PIO framework (population-intervention-outcome), inspired by a model used in pilot studies for evidence-based research in nursing ( Arguelles, 2011) ^1^. Based on the results of the pilot study, we developed the following review protocol:

***Time period***: 1990–2019. The time period was set to make an analysis of development over time possible while allowing only for literature available in the digital format.

***Literature included***: Peer-reviewed articles in English, available in the digital format.

***Databases***: Searches were conducted in two general databases, SCOPUS and Web of Science (Arts and Humanities Citation Index), and eight more specialised databases: Art & Architecture Source, provided by EBSCO, and Art Bibliographies Modern (ABM), Arts and Humanities Database, Design and Applied Arts (DAAI), ERIC, International Bibliography of Art (IBA), PsycINFO, and Sociological Abstracts, all provided by ProQuest.

***Keywords and search strings:*** Within the framework of PIO (population-intervention-outcome), "social exclusion" OR "minorities" OR "marginalised" were set as main keywords for population (with 12 keywords as subordinated variants), "performing arts" OR "visual arts" were set as main keywords for intervention (with 21 additional keywords as subordinated variants), and "social impact" OR "empowerment" OR "policymaking" OR "evaluation" were set as main keywords for outcome (with 25 keywords as subordinated variants, see Appendix 1 for a complete list of keywords). When conducting the searches in each database, the three sets of keywords were combined by using AND ( Table 1).

**Table 1.** Search terms according to the PIO model.

Population	Intervention	Outcome
*Main search terms:* social exclusion, marginalisation, minorities	*Main search terms:* performing arts, visual arts	*Main search terms:* social impact, empowerment, policy making, evaluation
Indigenous Native Immigrant Migrant Refugee Intercultural* Diversity Underserved Underprivileged Poverty Gender Children Young people	Drama Theatre Teater Museum Performing art Contemporary art Arts education Art intervention Artistic project Community art Socially engaged art Participatory art Arts activism Public art Civic art	Social effects Social change Social outcomes Wellbeing Well being Health Mental health Quality of life Inclusion Citizenship Civic engagement Civic participation Equity Social equality Values Attitudes Tolerance Resilience Empowerment Skill enhancement Evidence Measurement Analysis Assessment Democratic development

The search resulted in 11,764 hits in 10 databases, chosen for their appropriateness for the subject ( Table 2).

**Table 2.** Distribution of identified documents from each source.

Database(s)	N
Arts and architecture Source (EBSCO)	2,374
Arts and Humanities Citation Index (Web of Science)	974
SCOPUS	2,850
Various Proquest databases	5,566
**Total**	**11,764**

The removal of duplicates stemming from the combination of databases using DOI (1404 duplicates) and title (133 additional duplicates) resulted in a total amount of 10,227 unique documents.

Although care was taken in retrieving documents, we identified some quirks of the respective databases during the process. Firstly, although the time frame for the retrieval was bound to 1990–2019, some publications outside these limits were found. Ten articles had no registered year. Sixty documents were published in 2020, and five had a publication year between 1978 and 1988. We did not investigate the reason for this further but surmised that different databases use different ways of identifying the year in the search (i.e., publication year vs submission year, the existence of preprints, and the possibility of actual errors in the included databases. The data was included in the analysis since we deemed that the bias introduced by their inclusion would be negligible for the final analysis.

### Outlining the field: descriptive analysis

**Figure 1.**
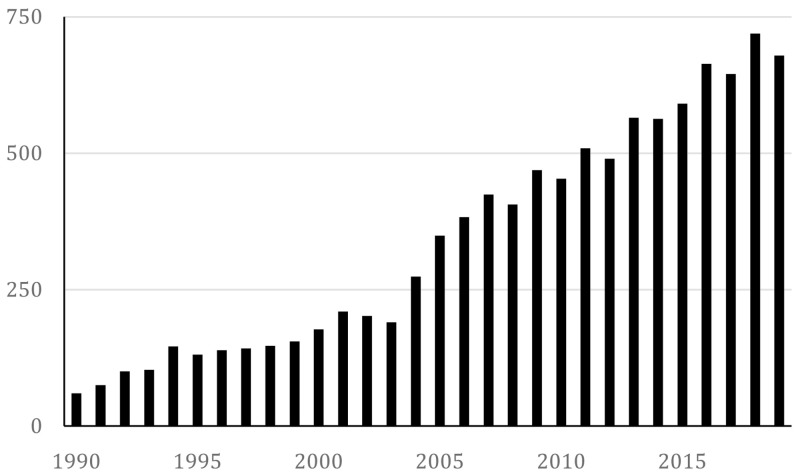
Number of retrieved documents yearly.

The bibliographic approach amounted to a description of the top 20 journals where the articles were published and a count of the number of articles published each year, summarised in Table 3 and Figure 1.

**Table 3.** Top 20 journals in the set.

Publication title	Count of final order
Arts Education Policy Review	210
Art Education	120
Research in Drama Education	119
Visual Arts Research	114
Studies in Art Education	98
Journal of Aesthetic Education	94
Curator	92
Museum International	89
New Theatre Quarterly	87
Youth Theatre Journal	82
International Journal of Art & Design Education	62
Journal of Museum Education	55
Journal of Archaeological Science	54
Theatre Topics	52
Theatre Survey	51
International Journal of Education & the Arts	51
Antiquity	50
Third Text	46
Museum Management & Curatorship	43
International Journal of Education through Art	41

From this list, we can see that the discipline of arts education is dominant in the field and that journals dedicated to research on theatre and museums are at the forefront. Among the top 10 journals, five are indexed in Web of Science. The other journals are indexed in Scopus, except Visual Arts Research, which is not represented in Journal Citation Reports or Scopus.

From this figure, we can derive that research interest in the social impact of the arts, interpreted as the number of articles published per year and operationalised through our search strings, have increased since 2005 and tripled in amount to around 600 in 2015. Thus, it is an expanding field, which further motivates the need for mapping trends in the data. 

### Analysing emerging themes in the data

**Figure 2.**
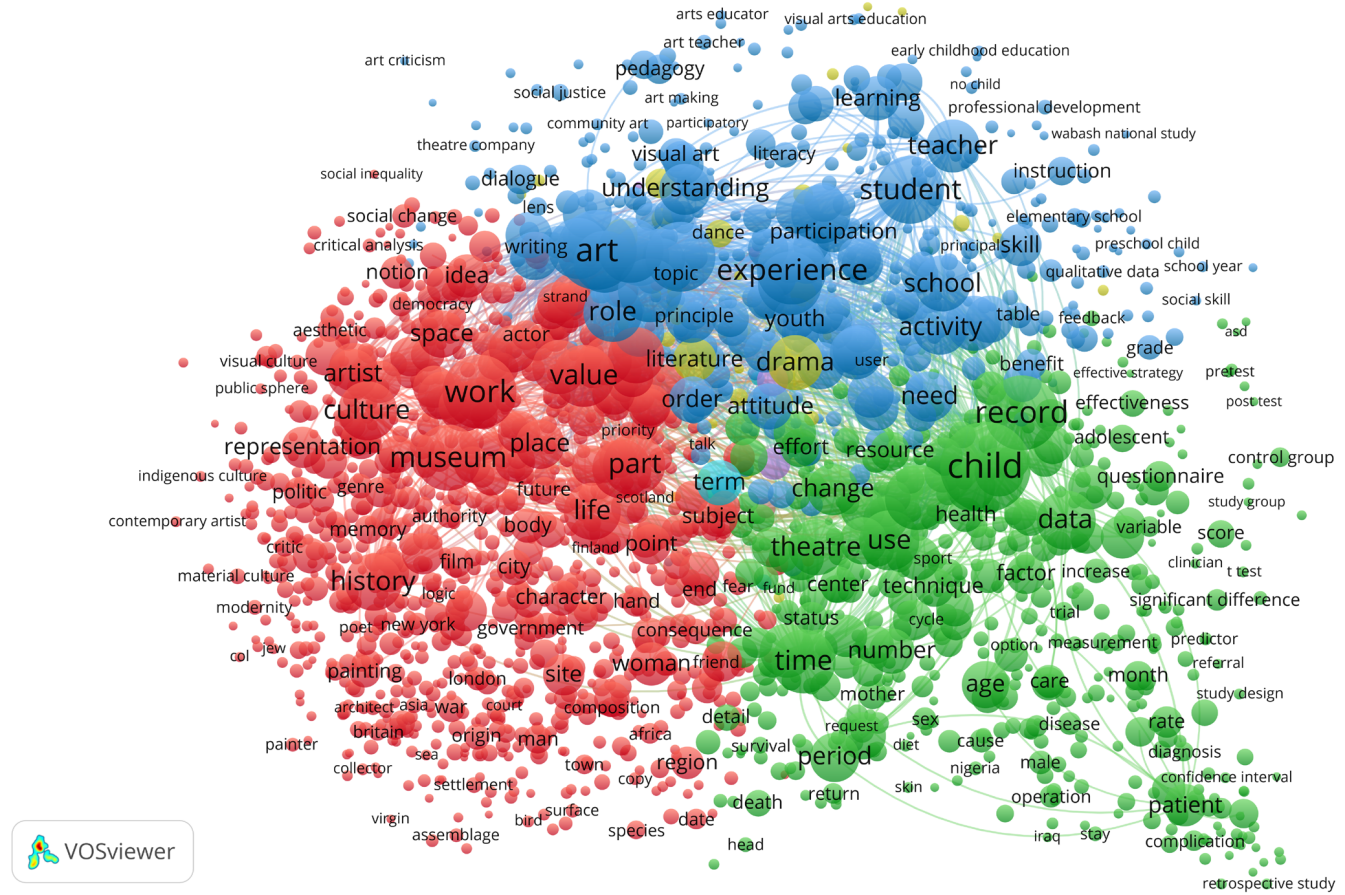
Co-word analysis of terms found in titles and abstracts in the data set. The co-occurrence of 1827 noun phrases found at least 20 times in the data set was used to construct the visualisation. Three main clusters were identified based on the thematic closeness of the terms in the texts.

We performed a text-based content analysis to understand emerging themes in the data, which used the terms found in the titles and abstracts of the collected publications to identify the topical structure of the data. Co-word analysis was used to identify topical clusters within the collected texts, consisting of terms often found co-located in the texts. Using the software package VOSviewer ( Van Eck & Waltman, 2010), an analysis of the contents of the titles and abstracts of the articles was performed and visualised as a co-word map. VOSviewer employs a method for "linguistic" parsing (the Apache OpenNLP programming library, *ibid*.) that identifies noun phrases, meaning words or combined terms including nouns or adjectives in front of nouns in a data set. We identified that the co-occurrence of these noun phrases calculated a distance measure based on the co-occurrence of each pair of noun phrases. Lastly, a threshold for inclusion in visualisation was applied, limited to noun phrases occurring at least 20 times in the text (n=1966). In our treatment of the data, we found that generic terms, such as article, author, and paper, obfuscated the results in the visualisation. Therefore, we performed a manual inspection of the noun phrases and removed these generic terms. The resulting data set that was used in the analysis consisted of 1827 terms. The visualisation of the terms used in the articles presented us with three overarching themes, demonstrated in three clusters ( Figure 2).

We named each cluster based on the topic areas of conversation related to artistic impact in the graph. Since the co-word analysis is calculated as the statistical relationship between all terms, there is an overlap between the categories. The boundaries between categories are more blurred than the colours indicate. This does not mean that the clusters cannot be distinguished from each other, but rather that terms are gradually more related with each other the closer they are to the boundary. The red and the green clusters are leaning on the blue one to suggest a correspondence between terms used in articles from all three clusters. For instance, terms like "attitude", "order", "change", "effort", "part", and "subject" link these three clusters together in the overarching themes of culture, learning, and health. An analysis based on the word occurrences in the cluster resulted in the below thematic identification:

**The red cluster** is mainly concerned with *issues of culture and society*, such as the value of culture within the geographical context of cities, countries, or regions. The terms within the red cluster consider aesthetic culture as well as anthropological culture in the broader sense. Culture is also understood concerning the museum as an institutional space and heritage institutions’ artistic and cultural work. Although "women" is a term in this cluster, it typically lacks a specified analytical subject.

**The blue cluster** thematises *issues of art and education*. It typically deals with the experience of learning in an institutionalised and formal setting or a more informal community setting. The typical research subject in this cluster is the pupil or, more broadly, the child.

Lastly, **the green cluster** connects *issues of art and wellbeing*. It is primarily focused on health issues in context, with methodologies and techniques for measuring the use of art to improve health and well-being among the subjects of intervention.

The visualisation allowed us to understand how these clusters represent various epistemological and theoretical traditions, overlapping but disparate enough to speak of them as subfields.

***Selecting articles for a qualitative analysis of the themes.*** To validate the thematic structure of the field, we did a qualitative reading of articles representative of each cluster based on the co-occurrence of terms in the visualisation. We used an approach based on the term weighting of the titles and abstracts to identify relevant articles to read. We wrote a script that determined the number of times a specific term was found in each text and a relevance measure that measures how distinctive the specific term is in the data set. Using the visualisation, we identified specific terms used for selecting relevant articles to read in the qualitative part of the study. We initially focused on the ten most common terms per cluster, five relating to the other clusters and five isolated terms in each cluster. The isolated terms were interpreted as those that set the theme apart, while the related themes provided articles that can be argued to belong to overlapping themes. This work resulted in 20 distinctive terms for each of the identified thematic clusters found in the visualisation. We identified 50 documents for each term and used the metadata consisting of title, journal information and DOI to determine the actual document to read manually.

Based on the three identifiable clusters generated by co-word analysis, the specific terms we used to identify documents were:

**Culture and society cluster**

Terms related to the other clusters: “ *value,” “part,” “idea,” “subject,” “country.”*

Terms isolated from the other clusters: “ *work,” “culture,” “museum,” “place,” “history.”*

**Art and education cluster**

Terms related to the other clusters: “ *experience,” “concept,” “community,” “activity,” “need.”*

Terms isolated from the other clusters: “ *pedagogy,” “understanding,'' ''student,” “teacher,” “learning.”*

**Art and well-being cluster**

Terms related to the other clusters: “ *record,” “child,” “change,” “theatre,” “time.*”

Terms isolated from the other clusters: “ *patient,” “treatment,” “age,” “data,” “use.”*

Provided that the research articles thematized art/culture, we strived to read the articles in dialogue with one another by posing the following guiding questions in the analysis: How do the articles conceive of the role or effect of the arts? How is this role or effect realised/explored in research? What conclusions do the articles wish to disseminate to the research community?

## Results: what research is typical for the themes?

### Art and society: theorising the changing role of the arts

The articles from the art and society cluster, especially those containing terms isolated from the other clusters, conceptualise the role of the arts and culture to reflect developments in society and foster shared values for social welfare. Methodologies are not fitted into the PIO model of experimental research but were philosophically/theoretically oriented, sometimes lacking empirical material. Epistemologically, research in this cluster broadly relates to the social sciences/humanities research.

Typical research themes are related to debating *the (changing) role of the arts* (e.g., Delacruz [2011], from the isolated term “work”). Belonging to the same theme is an exploration of *the changing role of cultural institutions*, such as museums and libraries (e.g., Burgess [2009], belonging to the isolated term “history”; Martens [2015], related to the isolated term “culture”). Drawing on the concept of “site” as a meaningful place or location, Morris & Cant (2006, belonging to “place” as an isolated term) critically examine the relation between art and places through observation of artistic practices. Thus, the isolated terms are found in studies that debate the role of arts and culture in a community context, not so much with a methodological focus as critically approaching the significance of artistic practices for society. Terms like “culture”, “history”, “work”, and “place” are also rather general, indicating less group-oriented or targeted interventions than interventions directed towards a general populace. 

The research of the related terms contrasts to the isolated terms through *a more apparent presence of methodological interest or exploration*, concerned, for instance, with patterns of and reasons for engagement in the arts among older people (55+) and barriers to such engagement. Based on survey data from the Taking Part survey of 2007, commissioned by the Department of Culture, Media and Sport together with Arts Council England and face-to-face interviews ( Keaney & Oskala [2007], belonging to “part” as a related term), analyses some of the current trends observed in the case of the elderly. According to the article, the diversity of this group has been considerable and is likely to increase, particularly as regards ethnicity. A better understanding of what motivates older people to engage with the arts is needed "to know where policy intervention can make the most difference" (p. 353). The article thus recommends longitudinal studies for the future to better understand trends and changes in patterns of arts engagement among older people.

Furthermore, there are issues of developing "methods which had the capacity to tell stories of coexistence on country, in a place where Aboriginal and non-Aboriginal people have been immersed in a cycle of relations for over 230 years" ( Harrison *et al.*, 2016, p. 1330, belonging to “country” as a related term). The method in this article may be understood as primarily defined by its context, where students are engaged in communicating with members of an indigenous population while producing a mural painting based on the narrative that emerges. Likewise, Chung & Ro's article (2004, belonging to the related term “subject”) studies the effects of problem-solving instruction in practical arts education on the creativity and self-efficacy of children. Finally, the effect of museums as cultural educators is critically debated by Earle (2013, belonging to “value” as a related term) by reference to the crisis of legitimacy that has been challenging museums since the end of the 20th century. Thus, what can be observed as to the related terms is an educational focus, such that there is a connection to the art and education cluster and its concern with the relation between art and learning.

### Art and education: teaching arts, art teaching

This cluster was thematised as relating to issues of arts/culture in connection with pedagogy/learning, especially as regards research belonging to the isolated terms. This cluster is exemplified by research on theatre pedagogy studied for intercultural education research ( Frimberger, 2016, Pedagogy as an isolated term). Such pedagogy is deemed to comprise an alternative to conventional paradigms of acting, in that young people are trained to theatrically explore their identities through the expressions of their bodies ( Tuisku, 2015). This cluster is also exemplified by articles on the aesthetic understanding of children and young people, where art education is believed to help students acquire knowledge and explain and apply concepts at different levels of cognitive development ( Lekue, 2015, “understanding” as an isolated concept). The study tests the hypothesis of statistical correlation between cultural understanding and academic goal orientation, engagement in art activities and attitudes towards art education in school. Articles on aesthetic means of learning also thematise technology or digital tools to trigger interest and self-confidence in students. For instance, Ho & Lin's article (2015, “learning” as an isolated term) article describes a technology-based art intervention and its impact on paper-based art creation and learning attitudes of students.

Research on teacher education and teacher subjectivity is explored concerning multiculturalism to examine the development of teacher educators concerning their commitments to educational equity and a critically informed teacher education ( Stillman & Beltramo, 2019, “teacher” as an isolated term). The role of multicultural art is discussed to promote pleasure and cultural skills and encourage the critical questioning of ideological representations of cultural identities ( Reisberg *et al.*, 2006). Articles are further directed towards the engagement with the surrounding community as incorporated in arts-based drama education. SmithBattle (2012, “student” as an isolated term) studies the pedagogy of student-created dramatic performances to promote reflection on sexuality and health care experiences. The role of theatre is understood to "foster empathy and convey the complexity of clinical situations from the vantage point of patients and clinicians'' ( SmithBattle, 2012, p. 591). In the case of Feagan & Rossiter (2011), a pedagogical experiment was carried out involving collaboration between a university and local community agencies. According to the authors, the method helped forge connections between community members who might not otherwise have encountered one another. The role or effect of theatre is enacted in these research articles by the evaluation of student assignments oriented towards team-based learning, in which students were to develop and perform dramas based on interviews with teenagers, thus getting to learn from first-hand accounts of real-life situations ( SmithBattle, 2012, p. 593).

As to the related and more general terms, there is a focus on investigating aesthetic experiences ( Woodward & Ellison, 2010, “experience” as a related term). There is a concern with the transformative potential of subject-object relations in the context of art ( Woodward & Ellison, 2010). The role or effect of art and art education, more specifically various forms of improvised drama, is studied by DeBettignies & Goldstein (2020, “concept” as a related term) as a means to improve the self-concept of children to promote their chances to succeed in school as well as in life in general. “Concept” refers to self-concept, a multifaceted construct of cognitive and physical abilities and social functioning, status, and recognition.

Addressing the future needs for qualitative, accessible, and appropriate housing for the elderly, is where the role of art, in this case, is finally opening channels of communication ( Bailey *et al.*, 2019, belonging to “need” as a related term). Through the collaborative use of a variety of visual and performative arts, the idea was to facilitate public conversations about growing older in homes, to raise important issues for those working in housing, care and health services, as well as to urge housing and health care policymakers to take the necessary measures for years to come. Activity appears in the context of joint art creation with clients with learning disabilities ( Furneaux-Blick, 2019, “activity” as a related term). Art therapy is further conceptualised to meet clients on their terms and address the power imbalance inherent in work with disabled children. Articles in this cluster can thus promote the significance of art education by taking it out of the classroom and putting it to the benefit of the community at large ( Ortiz & Chung, 2011, “community” as a related term).

The related terms of the art and education cluster are thus clearly related to the art and well-being cluster, and are targeted at the elderly, and disabled students. However, they are also directed towards experiences of art and culture in general. The isolated terms are more student-oriented and situated in a formal learning context, in contrast to related terms that form a bridge back to the art and society cluster and its focus on community welfare.

### Art and well-being: developing methods for understanding effects

Findings in this cluster (green) were discussed in tandem with theory, especially in research belonging to the isolated terms (for example, understandings of youth transitions theory and Piaget's developmental stages of the child). Research belonging to the isolated terms was found to be profession-oriented, such as insights into developing art therapy methods for autistic children ( Cao *et al.*, 2013, belonging to the isolated term “treatment”).

There is a typical research design in this cluster of combining a quantitative and qualitative approach with participatory involvement of the target group (conceived of as "peer researchers"), such as questionnaires, qualitative interviews, and participatory creative research workshops. Impact is thus an essential concept for research representing this cluster, for example, the impact of art on improved health and satisfaction outcomes on pediatric patients ( Nanda *et al.*, 2009, “age” as an isolated term). Mixed methods are reported, such as surveys and recorded qualitative comments for finding significant differences in art preferences across the different age groups, ( Nanda *et al.*, 2009) or using a mixed-method qualitative approach of questionnaires and focus group studies to approach film as a platform to engage, evoke and develop student nurses' understanding of the patient experience ( Ogston-Tuck *et al.*, 2016, “patient” as an isolated term). The impact of the art intervention was discussed concerning the way film can help nurses to respect the patient’s experience of disease, disability, death, and its reality.

Typically, the articles in the related terms of the art and well-being cluster showed a topical diversity again, compared to the isolated terms related to a medicinal research tradition. For example, research in this cluster belonging to the related term “change” involves developing tools for understanding short-term effects on young people's knowledge and attitude toward climate change though visiting a science centre exhibition in Finland (with the results of the study indicating that the exhibition experience did not support comprehensive attitude change, [ Gorr, 2014]). There were also studies aimed at providing researchers and practitioners who work with children with a model for creating guided-play activities in which children experience a range of emotions. Thus, according to the researchers, they develop social and emotional skills ( Goldstein, 2018, “time” as a related term). Other research was more user-oriented, focusing on the participatory element, such as Brahma, Pavarala, & Belavadi, (2019, belonging to the related term “record”) who studied the use of theatre targeting regressive social norms relating to gender inequality, such as domestic violence, child marriage, women’s and children's health issues.

Research in this cluster is exemplified by the need to articulate the aims and objectives of cultural provision and generate 'evidence', such as the impact of youth theatre for non-arts audiences ( Hughes & Wilson, 2004, belonging to “theatre” as a related term).

### Comparing the clusters

The thematic analysis confirms that the art and society cluster represent research that takes a more generalised take on the role of arts and culture, in contrast to research with a greater focus on certain target groups (such as children and young people and their developmental abilities, behaviours, or attitudes). The articles tend to offer a reflective narrative, compared to traditional scholarly texts where specific theoretical assumptions are set against empirical material and conclusions are drawn (although such research exists). The tendency to include more general social developments as a theme makes this cluster reflect a scholarly narrative from the humanities rather than the social sciences or natural sciences. The term “subject” refers to the art and well-being cluster in being concerned with child development, whereas “value” relates to the isolated term “museum” in the art and society cluster. Part may also relate to the lifecycle (birth, death...) of the art and well-being cluster, in that the health conditions of older people are taken into account as regards their engagement with the arts. Finally, we would say that “country” links together the clusters in the sense that it is about community art and learning, and a connection to the lifecycle of people and places.

Research in the art and education cluster is characterised by a strong belief in the significance of the arts and its impact, mostly as a learning tool, and are often set out to show how this played out in different projects. In the articles studied, it is indicated that the relation between teacher and student is more of an agreement than one characterised by a power imbalance. The teacher is not so much in focus as the student-actor or spectator. Hence, pedagogy has more in common with art-making, although studied from the perspective of art educators and the de-centring of art education. The concept of activity thus relates again to the art and well-being cluster of children's development and health. Understanding through art is conceived of the visual component in learning and has connections to self-expression. Understanding, literacy, and psychology are close because it is about the child's psychological and sociocultural development concerning aesthetic and conceptual abilities to articulate or represent knowledge. The related terms, such as “activity”, are close to the art and well-being cluster and take an art-therapeutic approach. One important conclusion is that the concept of “community” in this cluster is participation oriented. Research belonging to the term aims to show the value of art education for communities at large and how it can contribute to the lives of community members.

Research typical for the art and well-being cluster, both in terms of belonging to terms isolated from and related to the other clusters, encompasses topics related to the understanding of how various aspects of art, in various settings and context, can impact a specified set of targeted populations, whether nurses, children in pediatric care, women from a specific community, or school children. Thus, this cluster represents an experimental theme focused on methodologies to capture the effects of arts interventions. The more isolated the term, the more this focus on methods relates to a medical field, but the cluster is not restricted to studies of the impact of the arts in medical research. As studies involving children are common in this cluster, ethical issues regarding interventions for children demand rigorous methodologies for conducting and capturing the effects of the arts and culture.

In sum, these clusters overlap in epistemologies but are distinguishable in their focus. Roughly, these themes present art’s social impact relating to health and wellbeing, education, and knowledge (or cognitive learning skills), and less identifiable, community and identity. The child, often in formal learning contexts, is found chiefly as the targeted population (where the PIO-categories are distinguishable), theatre/drama is the most common intervention, and knowledge/skills enhancement is the most common outcome.

## Mapping the research front

Articles with similar references are related content-wise. This is sometimes labelled a “research front” ( Persson, 1994) in bibliometric terminology and builds on the idea that articles that cite similar references carry the “symbolic content” ( Small, 1978) of the cited works. Understanding the research front is sometimes used to determine the “best research,” however, we were interested in understanding emerging themes in such a set of articles, to map how the field forms related content.

We developed a combined bibliometric and semantic method based on the bibliographic coupling of articles, as quantified by their shared references, to generate a relevant set to analyse manually. Using the VOSviewer software (see van Eck & Waltman, 2010), we analysed a subset of 974 articles (W) part of the set of initially identified articles (A), obtained from Web of Science, to produce another cluster analysis of the documents to categorise them into thematic parts. This time, the relatedness of individual articles (identified as ‘author’, ‘year’ in the graph) was based on shared references in their respective reference lists. A content analysis of frequently cited articles in the clusters was performed to extend the analysis from these articles in *W* to the full set *A*.

**Figure 3.**
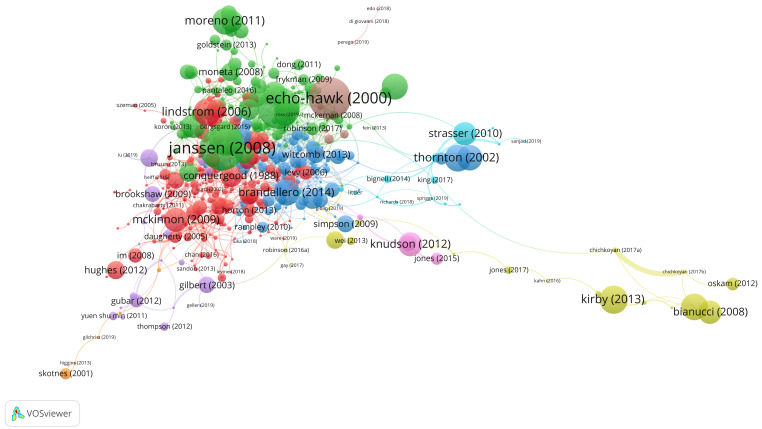
Bibliographic coupling of articles as quantified by their shared references.

The following image ( Figure 3) visualises the mapping of nine clusters with regards to the most cited articles. This overview tells us how authors relate their work to other works to form themes of research. In the paragraph below, we will discuss these themes and their relevance for the analysis of the research front.

### About the clusters

After forming an overview of the nine different clusters or areas of the bibliographic coupling visualisation above, considering the titles of articles and their thematic content, we found that the red and green clusters were most interesting for a qualitative analysis of the research front regarding the art’s social impact. Based on the criteria explained below, we did not examine all of the clusters in this dataset, partly to help us define a set of articles for analysis that was manageable within the set time frame of writing (two researchers, Lindström Sol and Gustrén, were responsible for the qualitative analysis of reading the articles).

For example, although the blue cluster (n=99) contained a few interesting contributions regarding the work of cultural institutions, we found several instances in this cluster thematising analysis of objects (e.g., metallurgic, jewellry). Since this was not within our chosen scope, we excluded this cluster from further analysis.

Moreover, the yellow (n=23), orange (n=6) and brown (n=4) clusters mainly contained works on topics like archaeology, biology, palaeontology, and other domains within the natural sciences as well as history, prehistory, and antiquity. Ethnographic approaches to the ancient world and its oral traditions were a recurrent feature in these clusters. For instance, Echo-Hawke (2000) was a highly cited article in the orange cluster, whose title and abstract indicates that the article is tracing oral traditions dating back to ancient history. Although interesting, we did not deem it relevant enough for a study on the social impact of the arts from a contemporary perspective.

The same goes for the purple cluster (n=16), mainly dealing with childhood, nursery, play and similar features. The turquoise cluster (n=6) was about Elizabethan theatre, especially Shakespeare. Again, no indications of any effects were considered, even if such effects may be indirectly discernible in the studies. Finally, the pink cluster (n=3) was about various forms of audio description or studies of sound in the context of theatre and museum exhibits. These articles were too few and lacked citations, which led us to consider them too marginal to identify a potential research front.

Thus, for the analysis of the research front on the arts social impact, we performed a qualitative reading of the top-cited articles (operationalised as those with ten or more citations) in the remaining clusters, which we defined as the research front of the social impact of the arts ( Table 4).

**Table 4.** Articles and no. of citations in the analysed clusters.

Red cluster (n=21 of 275)	C (>10)	Green cluster (n=23 of 130)	C (>10)
Lindstrom, 2006	*40*	Janssen *et al.*, 2008	*107*
McKinnon, 2009	*31*	Ash, 2003	*94*
Conquergood, 1988	*25*	Neelands, 2009	*59*
Vannini & Waskul, 2006	*21*	Moreno *et al.*, 2011	*38*
Campbell, 2008	*20*	José del barrio *et al.*, 2009	*36*
Edmondson, 2005	*20*	Peppler, 2010	*31*
Gregory, 2003	*19*	Day, 2002	*26*
Dawson, 2018	*19*	Leonard, 2010	*26*
Hughes, 2012	*18*	Rickard *et al.*, 2012	*26*
Heddon *et al.*, 2012	*15*	Göncü & Perone, 2005	*23*
Henriksen & Frøyland, 2000	*14*	Adams, 2017	*19*
Miller, 2008	*13*	Balloffet *et al.*, 2014	*18*
Lum & Dairianathan, 2014	*12*	Moneta & Rousseau, 2008	*18*
Tofteng & Husted, 2011	*12*	Schuele & Lederman, 2004	*14*
Wake, 2013	*12*	Lauring *et al.*, 2016	*13*
Bernard, 2014	*11*	Eerola & Eerola, 2014	*12*
Im, 2008	*11*	Gibson, 2003	*12*
Levy, 2006	*11*	Greenfader *et al.*, 2015	*12*
Page *et al.*, 2006	*11*	Sapiro, 2015	*12*
Taylor, 2013	*11*	Hsiao, 2010	*10*
Horton & Berlo, 2013	*10*	Octobre, 1999	*10*
		Sonn *et al.*, 2015	*10*
		Wheeler, 2003	*10*

### Analysing the research front: topical themes

From reading the titles, abstracts, and the articles (when the abstract was unclear as to population, intervention, outcome), we did not deem the two remaining clusters disparate enough for a meaningful separation of topical analysis, except for one account. Although largely overlapping, the red cluster tends to thematise informal learning settings such as theatre/drama as an intervention. In contrast, the green cluster tends to thematise formal educational settings such as schools and museums.

In our reading, we used the PIO model, i.e., defining population, intervention, and outcomes in the articles, to form a basis on which we can discuss the understanding of the social impact of the arts. From a reading of the titles and abstracts of the most cited articles in the chosen clusters (n=44), we excluded two articles on the following grounds: the article was not written in English ( Octobre, 1999), and the article did not consider the arts/culture as a theme ( McKinnon, 2009), instead considering performing vulnerability in the courtroom; it is therefore clear our method is not sophisticated enough to differentiate between figurative uses of ‘performance’. Overall, we analysed 42 articles in order to understand the scope of the research front of art’s social impact.

## Results: an overview of most common populations, interventions, and outcomes in the research front

The included articles were analysed through an in-depth reading to outline the population, intervention, and outcomes, both intended and observed. This presented us with a few interpretational difficulties since most articles were not written within the PIO framework. To identify population, we set out to understand the effects of arts/culture on something or someone, and this something or someone was interpreted as the population. Instead of a defined group of people as the population, it could be more abstract concepts such as arts journalism, or political developments and/or the perception of a country or region through art, or as we will discuss in more length later, the effects of political, geographical developments on art.

When identifying interventions, we struggled, for example, with the question of what is to be understood as intervention in studies of theatre plays - the analysis of the play or the actual play? In some of the articles, the author was not part of an investigation of an artistic intervention but engaged in an analysis of cultural products intended to theorise about their significance in various ways. We chose to define intervention as the observed artistic/cultural product/process. We also decided to differentiate between intended and observed outcomes, where the emphasis was placed on the latter.

Interventions were more straightforward as points of analysis. Our analysis formed several categories of observed outcomes that will be discussed for understanding the research front on the social effects of the arts.

### Populations, most articles related

The results show that the most common population is children and young people in formal learning settings, and theatre/drama being one of the most common interventions. This confirms that the research front follows the theme of the co-word analysis, where these categories were also common in the overall data. The second category, unspecific or broadly defined populations, related largely to the potential of cultural institutions, such as museums, in terms of learning. Thus, this analysis concludes that parts of the research front on the social impact of art, as defined by our search methods, relate the importance of social impact to the relevance of cultural institutions. Marginalisation issues, such as minorities and refugees, are not the most prominent themes in terms of populations in research ( Table 5).

**Table 5.** Themes belonging to population in the data analysed.

Populations	Empirical instances
**Children and young people in formal and** **informal learning settings** **(15 articles)**	Children without earlier music training ( Moreno *et al.*, 2011) Primary school child, musical learner? ( Lum & Dairianathan, 2014) Young people/underprivileged youth ( Lindström, 2006; Neelands, 2009; Peppler, 2010) Pupils, children 3, 6, and 12 years old ( Eerola & Eerola, 2014) English learners in the primary grades ( Greenfader *et al.*, 2015) Teacher students ( Page *et al.*, 2006; Gibson, 2003) Art naïve uni students ( Lauring *et al.*, 2016) Primary and secondary school students ( Rickard *et al.*, 2012; Day, 2002) 20 children aged 4–5 ( Hsiao, 2010) Former child soldiers of post-war Northern Uganda ( Edmondson, 2005) Young audiences ( Balloffet *et al.*, 2014)
**Unspecific/broadly defined populations** **(10 articles)**	Individuals ( Vannini & Waskul, 2006) Diverse communities ( Campbell, 2008) The audience participant ( Gregory, 2003; Heddon *et al.*, 2012; Henriksen & Frøyland, 2000) Interpretative communities ( Miller, 2008) Arts journalism ( Janssen *et al.*, 2008) Families ( Ash, 2003) Visitors ( Leonard, 2010) Adults ( Göncü & Perone, 2005)
**Professionals within the culture sector** **(5 articles)**	Regional heritage institutions ( José del barrio *et al.*, 2009) Performers and directors ( Taylor, 2013) Publishers and translators ( Sapiro, 2015) Musicians ( Schuele & Lederman, 2004) Artists and venue providers ( Wheeler, 2003)
**Political geographies** **(4 articles)**	The Israel-Palestine conflict ( Bernard, 2014) Post-colonial Korean society ( Im, 2008) The nuclear in post-war Britain ( Hughes, 2012) Post-colonial Uzbekistan ( Adams, 2017)
**Indigenous, minority people** **(4 articles)**	Indigenous artists, Native North American ( Horton & Berlo, 2013) Sami people ( Levy, 2006) Aboriginal and non-indigenous children, young people, adults in four rural towns ( Sonn *et al.*, 2015) Participants from low-income, minority ethnic backgrounds ( Dawson, 2018)
**Immigrants and refugees** **(3 articles)**	Hmong refugees ( Conquergood, 1988) Asylum seekers ( Wake, 2013) Immigrant adolescents with behavioral difficulties ( Moneta & Rousseau, 2008)
**Disadvantaged (1 article)**	Unemployed people ( Tofteng & Husted, 2011)

### Interventions, most articles related

The most common interventions confirm the co-word analysis where theatre/drama, in formal and informal educational settings, dominate the research front. Music and visual arts are also common, along with museum activities ( Table 6). The category “media and popular culture” relates to the outcome category “theory/method development”, which will be elaborated on in the next section.

**Table 6.** Themes belonging to intervention in the data analysed.

Interventions	Empirical instances
**Art education/formal learning settings** **(12 articles)**	Student portfolios ( Lindström, 2006) Music/visual arts education ( Lum & Dairianathan, 2014) Drama in schools ( Neelands, 2009) Effect of music training ( Moreno *et al.*, 2011) Use of new technologies for communication as part of arts curriculum ( Peppler, 2010) Forum theatre workshop in school ( Day, 2002) School-based music and visual arts instruction ( Rickard *et al.*, 2012) School based drama intervention ( Moneta & Rousseau, 2008) Extended music curricular class ( Eerola & Eerola, 2014) Attitudes to art and art education ( Gibson, 2003) Drama and creative movement intervention among students ( Greenfader *et al.*, 2015) Art-making activities among kindergarten children ( Hsiao, 2010)
**Theatre** **(11 articles)**	Health theatre ( Conquergood, 1988) Shared memories through drama ( Campbell, 2008) Drama as arts therapy ( Edmondson, 2005) One-to-one theatre performance dialogue and collaboration ( Heddon *et al.*, 2012) Theatre-based action research ( Tofteng & Husted, 2011) Verbatim or testimonial theatre ( Wake, 2013) Political theatre ( Bernard, 2014) Intercultural theatre in Hamlet ( Im, 2008) Verbatim theatre ( Taylor, 2013) Adult improvisation based on child play ( Göncü & Perone, 2005) European style theatre ( Adams, 2017)
**Media and popular culture** **(7 articles)**	Life-from-space theory in popular culture ( Gregory, 2003) Science communication ( Dawson, 2018) Popular imagery and culture of the nuclear ( Hughes, 2012) Digital gameplay ( Miller, 2008) Analysis of international arts and culture coverage ( Janssen *et al.*, 2008) Dissemination of French literature ( Sapiro, 2015) Institutionalization of performance art ( Wheeler, 2003)
**Museum activities** **(6 articles)**	Museums for scientific literacy ( Henriksen & Frøyland, 2000) Exhibition analysis of the representation of Sami identity ( Levy, 2006) Families making sense of science content in museums ( Ash, 2003) Techniques for efficiency evaluation in museum management ( José del Barrio *et al.*, 2009) Exhibitions on popular music ( Leonard, 2010) Edutainment within the museum sector ( Balloffet *et al.*, 2014)
**Visual arts** **(4 articles)**	Attitudes to contemporary, socially orientated art ( Page *et al.*, 2006) Contemporary arts discourse on indigenous understandings ( Horton & Berlo, 2013) Art appreciation ( Lauring *et al.*, 2016) Photography and photo elicitation ( Sonn *et al.*, 2015)
**Music** **(2 articles)**	Aesthetic experience of life through music ( Vannini & Waskul, 2006), Review on occupational disorders of instrumental musicians ( Schuele & Lederman, 2004)

### Outcomes, most articles related

The most common theme regarding outcomes can be argued to belong to an internal, academic debate, furthering conceptual and theoretical knowledge on the social impact of the arts. The prominent themes of skills enhancement and knowledge dissemination/learning mirror the co-word analysis result, where the social impact of the arts in terms of outcomes are conceptually related to learning outcomes ( Table 7).

**Table 7.** Themes belonging to outcomes in the data analysed.

Category	Author/year of publication	Intended outcome/aim	Observed outcome(s)
**Theory/method** **development** **(8 articles)**	Gregory, 2003	Study of assessment tool’s ability to capture a scientific theory’s popularisation via popular press, museums, etc.	The paper argues for science communication studies to look beyond traditional categories to embrace the wide variety of media and genres that contribute to the construction of science in the public.
	Vannini & Waskul, 2006	In this article, the authors argue for the creation of the metaphor life as music.	From within the metaphor of life as music, the authors conceptualize beauty as the diverse rhythms, melodies, and harmonies contributing to the constitution of both subjectivity and intersubjectivity.
	Miller, 2008	This article investigates the *Grand Theft* Auto video game series in order to demonstrate the potential of a folkloristic, ethnographic approach for the analysis of digital games.	This case study suggests that digital gameplay should be regarded as a form of performance practice with the capacity to invoke traditional folkloric genres and engender new traditions.
	Campbell, 2008	A discussion of artistic projects concerning sharing the past. Theories of memory work, memory sharing as a cultural practise.	Shared experiences “allows us to forge a usable past together.”
	Neelands, 2009	Discussing the ensemble-based approach of drama in schools. Contrasts the pro-social emphasis in the ensemble model with a pro-technical emphasis.	Using ideas drawn from McGrath and Castoriadis, the paper claims that the ensemble approach provides young people with a model of democratic living.
	Hughes, 2012	Effects of cultural interpretations of a phenomenon (nuclear culture) on popular imagery.	Risk of homogeneous storytelling, reduction of pluralistic histories.
	Horton & Berlo, 2013	An analysis of why it has been difficult for the ‘new materialisms’ to incorporate indigenous intellectual traditions into discussions of non-human agency, focusing on contemporary arts discourse.	Indigenous artists’ understandings of material have an acute awareness of the contemporary, global challenges of cohabitation.
	Taylor 2013	This article analyses how affective listening is used to develop performances in Alecky Blythe's verbatim theatre.	The article argues that voice in *London Road* both claims and defers authenticity and authority, since voice signifies presence and embodied identity but the reworking of speech into song signals the absence of the real.
**Skills enhancement** **(7 articles)**	Lindstrom, 2006	Can the arts be assessed and taught? A study of portfolios as methods for art skills enhancement among young people.	The notion that assessments of learning outcomes must be either limited to superficial knowledge or completely arbitrary is shown to be a misconception. Improved visual design and artistic skills can be done in certain circumstances.
	Moreno *et al.*, 2011	The effect of 20 days of music or visual arts training on children’s pre-literacy skills.	(The children’s) “ability to map unfamiliar symbols to known words improved significantly from pretest to posttest” (p. 170). The effects on the music group were stronger.
	Greenfader *et al.*, 2015.	Effect of a Performing Arts program on the oral language Skills of young English learners	The treatment group ( *N* = 902) outperformed controls ( *N* = 4,338) on speaking assessments. Effects strongest on English learners with the most limited English-speaking abilities.
	Hsiao, 2010	Investigate methods of enhancing kindergarteners' artistic creative thinking and expressive drawing through appreciation of picture books.	Significant and positive change in children's reading and drawing behaviors at home. “The results also showed that the collage series of picture books had more impact on children than did other picture books in terms of teaching efficacy by picture book appreciation.”
	Eerola & Eerola, 2014	Can music education create social benefits in the school environment (general satisfaction about the school and a sense of achievement and opportunity for students)?	Extended music education enhanced the quality of school life and had a positive effect on the social aspects of schooling.
	Rickard *et al.*, 2012.	The impact of an increase in school-based music training on a range of cognitive and psychosocial measures for 10–13-year-olds in two independent studies.	No convincing benefits of school music classes were apparent. “The intrinsic value of music education for enjoyment and learning should therefore remain central to the justification of music education in the national school curriculum” (abstract).
	Moneta & Rousseau, 2008	Emotional regulation (ER) capabilities through drama intervention, immigrant adolescents with behavioral difficulties.	Some impairment in emotional expression and emotional regulation in this study sample. “In general, the drama process seemed to help emotional expression and awareness and to foster a transformation of emotive processes in the sense of a ‘collective ER.’”
**Knowledge** **dissemination/** **learning** **(7 articles)**	Henriksen & Frøyland, 2000	Explore “the potential of museums to provide information and experiences that the audience finds relevant in the context of science-related issues they encounter in their private or civic lives.”	Skeptical attitudes among families and museum professionals towards the role of museums for scientific literacy. No found effects threaten the relevance of museums.
	Ash, 2003	The article studies the effects of museums and museum pedagogy on families' scientific sense-making and learning.	Themes in knowledge arise from both the family members and the museum exhibit.
	Peppler, 2010	Understanding contribution of media arts education in informal settings to learning outcomes among underprivileged youth.	Using new types of software, young people can engage with technology that encourages active learning.
	Tofteng & Husted, 2011	How drama can empower action research processes in the field of unemployment. The article also discusses the reactions of the audience, “to use the plays to show others how life is outside the labor market.” (p. 37).	Theatre-based action research opens a new way to communicate and make visible knowledge and experiences from below that have difficulties reaching the public agenda or influencing structures of power.
	Levy, 2006	Examines the variable representation of Saami prehistory in several Nordic museums.	The presentation of Saami prehistory differs significantly between majority community museums and those run by Saami communities. ”The national and regional museums diminish or even deny a Saami role in the antiquity of the nation. In contrast, the Saami institutions grant the Saami the same ancientness as the other Nordic populations” (p 143).
	Dawson, 2018	Explore science communication (through popular media and cultural institutions) from the perspective of participants from low-income, minority ethnic backgrounds.	“Social reproduction in science communication constructs a narrow public that reflects the shape, values and practices of dominant groups, at the expense of the marginalised.”
	Leonard, 2010	Studies the effect of museum and museum pedagogy on audiences' sense-making and learning around popular music.	The exhibition “moved beyond the expected to represent a greater diversity of music genres, sounds, performers and dimensions. Value judgements always inform which exhibition ideas are given the green light and shape the way stories are told within displays” (p. 180).
**The impacts of the** **social on art/** **culture** **(7 articles)**	Lauring *et al.*, 2016	Impact of social and monetary contextual information on liking ratings of art.	Paintings with high monetary primes or with high ratings by peers and art experts led to higher participant liking ratings. In contrast, paintings with a low rating by the low-education/income social group led to higher liking ratings by participants. These results provide empirical support for the social “distinction” behavior theory.
	Im, 2008	This essay examines the implication of interculturalism in New Asia and ultimately the relationship between West and East, focusing on Lee Yountaek's production of *Hamlet* that premiered in Seoul in 1996.	Lee Yountaek's Shakespeare reflects the impasse of contemporary Korean society, whose postcolonial reality is obscured by an optimistic idea of interculturalism.
	Balloffet *et al.*, 2014	This article examines the concept of "edutainment" within the museum sector.	Edutainment presents both opportunities and risks: attracting new audiences, particularly young people, but also “Disneyfication” of cultural institutions.
	Janssen *et al.*, 2008	Studies the importance of national context and global hierarchies for arts journalism and the coverage of non-western culture/arts.	International coverage remains concentrated on a few countries, of which the United States has become the most prominent. Although the global diversity of coverage has increased, non- Western countries are still underrepresented.
	Adams, 2017	Examines the cultural change in Uzbekistan through the evolution of European-style theater during the twentieth century.	The article argues that the adoption of this theatrical form was part of a broader project of cultural modernization (...) “an example of a colonial hierarchy of cultures, which deemed European forms to be more advanced than indigenous ones. This orientation makes an investment in indigenous cultural forms less desirable since they are only intelligible on a local level.” (abstract)
	Sapiro, 2015	An empirical study of the circulation of French literature in the United States in the era of globalization.	Upmarket genres like poetry and theatre are better represented than commercial genres. The high centralization of the publishing field in the Francophone area impacts the circulation pattern. A by-product of the stiffening of commercial constraints on the publishing industry, the discourse on the ‘death of French literature’ paradoxically contributes to nourishing the well-founded fiction of national literatures.
	Wheeler, 2003	This article discusses the role of meaning in the institutionalization of performance art between 1970 and 2000.	“The process of institutionalization is shown as a paradox for American avant-garde art” (...) “Thus institutionalization is a process of negotiation shaped by meaning as well as social structure.” (abstract)
**Fostering ethicality** **and moral behavior,** **audiences and** **participants** **(5 articles)**	Bernard, 2014	Discussion of plays thematizing the Israel/Palestine conflict, examining controversies these plays engendered, and the effort to generate empathy and humanitarian feelings, to persuade a viewer to affiliate with a particular struggle or set of beliefs, and to commit herself or himself to action.	(These plays) “remind us that there is no specific politics associated with an empathetic response (...)” Perhaps these plays will persuade new audiences to ask themselves, with Hare, “Are we where we live? Or are we what we think?” (1998, 43, in Bernard, p. 172). Negative media reception.
	Day, 2002	This study investigates the experiences of, and interactions between, participants of a Forum theatre workshop, which addressed the issue of the refugee child at school.	Findings revealed that the workshop was highly relevant to the students, reflecting moral dilemmas which they faced in their everyday lives, as they encountered refugee students at school. This interactive workshop gave them the opportunity to try out moral behaviour, which could potentially be applied to real-life situations.
	Wake, 2013	A study of audience and media reception, and political effects of Australian play about asylum seekers.	Discussion on potential for pain and exploitation. testimonial theatre. “(...) while *Through the Wire* was ethically problematic, it was also politically efficacious” (p. 117). “The play was highly visible in the mainstream media. The realist aesthetic facilitated identification with asylum seekers (ibid.)
	Heddon *et al.*, 2012	A study of effects of one-on-one theatre performance, dialogue and collaboration.	Risk of intervention: the experiential performance proffers hierarchies of experience; invoking the notion of an ‘ideal audience-participant.’ However, also potential to produce more intimate connections, create togetherness in shared experiences.
**Assessment/** **evaluation** **(5 articles)**	Page *et al.*, 2006	Socially orientated contemporary art is making a success in the wider global context, but is omitted as practice in many schools, although the UK government recommends art as part of curriculum.	“Conclusions have been tentatively drawn about how the curriculum may be better served by the use of contemporary art, as well as the means by which new learning methods may be facilitated” (abstract).
	Lum & Dairianathan, 2014	Assess and evaluate the matching of policy and practice, ambition to include arts education, primarily music and visual art, as one of the key areas of focus in pursuing the goal of holistic education of a primary school child in the Singapore school system.	Survey research carried out in music education involving musical learnings from early childhood through tertiary education with a view to identify key areas of research interests and gaps.
	José del barrio *et al.* (2009)	Using a multivariate statistical technique to synthesise the initial information and data envelopment analysis (DEA) for efficiency evaluation in regional heritage institutions in Spain.	Promoting evaluation tools and methods for management of heritage institutions and public resource allocation.
	Schuele & Lederman, 2004	A study of common work-related injuries among instrumental musicians, assessing the risk for long- term disability.	Many of the medical problems encountered in instrumental musicians seem to have a good outcome and rarely lead to long- term disability except for focal dystonia. Work-related disorders might be eligible for health and wage-loss benefits through workers' compensation and private and state-funded disability insurance.
	Gibson, 2003	What do student teachers really think about art and art education?	Student teachers' prior experiences, existing knowledge, beliefs, attitudes, perceptions, and interest in the visual arts impacts the prospects of them adding visual arts education to their teaching in a primary school context.
**Health/** **well-being** **(2 articles)**	Conquergood, 1988	The effects of an environmental health education program, which employed performances and popular theatre, on critical awareness about the health problems: 1. among refugees in Ban Vinai, dealing with trauma and crisis, and 2. implications for medical health officials.	For health care programs to be delivered successfully, agency workers depend on the acceptance and cooperation of the recipients. A result of the study pointed to the need for more consciousness-raising activities for health professionals themselves. “For popular theatre to work effectively as a tool of critical awareness and empowerment for oppressed peoples it must be rooted in and begin with their cultural strengths” (p. 181).
	Edmondson, 2005	The study “explores the ways in which theatre and performance are used to market trauma and humanitarianism in northern Uganda.”	Arts therapy in northern Uganda was valued primarily to market trauma. “These works did not function as personal expressions of trauma and healing; instead, they were assimilated into the master narrative of war.” (p. 457). “In the creation of the World Vision plays, the staging methods worked to silence the children's voices, as their own ideas about children's rights or peacemaking were never solicited. Instead of speaking as commentators on war, they served as the mouthpieces for predetermined messages (p. 461).
**Community** **empowerment/** **identity** **(1 article)**	Sonn *et al.*, 2015	‘Voices’ used photography and photo elicitation as the medium for exploring and expressing a sense of place among aboriginal and non-indigenous children, young people and adults in four rural towns.	(The method) “reflected individual and collective constructions of place, based on positive experiences and emotions tied to the natural environment and features of the built environment” (...) “it is an approach that can contribute to community psychology’s empowerment agenda.” (abstract)

## Mapping the research front: discussion

The results reveal various interpretations of the arts’ social impacts that can inform researchers in this field. Again, the categories sometimes overlap. The analysis also found ambiguous, negative, or null results ( Henriksen & Frøyland, 2000; Rickard *et al.*, 2012), which are interesting for an understanding of the social effects of art.

Arguably, the results speak of the field as research that thematises more than traditional understandings of art. Instead, these encompass studies on cultural institutions, education, and popular culture. Secondly, it is worth asking if these results reflect social effects of the arts, or rather individual or group effects? Few articles in the data theorise the links between the micro, meso, and macro levels of impacts, and either do not take them into account or take them as given. For example, Henriksen & Frøyland’s (2000) study on the importance of museums for science-related issues for the public is examined through a group of parents, who are considered to be representative of the wider public.

When claiming effects in the dataset of articles, which is especially evident when claiming empathic responses from the audiences of theatre plays, the populations are not surveyed, nor are these effects tested (c.f. Bernard, 2014; Wake, 2013). Rather, these effects are taken as natural outcomes of the play’s structure: “When audiences think about where Rachel stood, until the moment that she stood in front of a bulldozer, they must also think about where they stand themselves.” ( Bernard, 2014: 170). This study was not intended to be evaluative, but we sometimes struggled to find evidence for the arguments for these effects (c.f. Daykin *et al.*, 2008). Studies on the effects of theatre participants ( Day, 2002; Heddon *et al.*, 2012) differ from studies of effects on audiences, with a methodology that allows for a discussion of tested results. Daykin *et al.* (2008:261) conclude that it is unlikely that a single method can be found that will serve as a “gold standard” in research on the impact of the arts. Although methods of capturing the impact of the arts are not straightforward, analysing the most cited articles in the data set is not the same as analysing “the best” research in the field in terms of clarity.

The articles aiming towards the theory or method development of their field have the least to add to a discussion on the social impact of the arts, as they focus on advancing their field. Neither do the articles analysed as assessment/evaluation of arts/cultural policy or institutional/educational strategies, as they take the meaning of art, often as a positive force, as given. When effects are accounted for, such as in providing young people with a model for a democratic living ( Neelands, 2009) or creating awareness of global challenges ( Horton & Berlo, 2013), these are not claimed but discussed as *possible* outcomes. For example, Schuele & Lederman’s (2004) study of common work-related injuries among instrumental musicians leads to an interesting discussion on musician’s eligibility for wage-loss benefits and state-funded disability insurances, which arguably would (if they were enforced) be a form of social effect. The articles in the categories “theory/method development” and “assessment/evaluation” make up ⅓ of the total articles, which casts doubt on this selection of data’s ability to tell us something substantial about the social effects of the arts.

When articles in the research field thematise identifiable social effects as something more comprehensive than the immediate effect of an observed, limited population (often children in a school setting), they can be listed as the following:

- Reaching the public agenda, achieving media response, or influencing structures of power ( Bernard, 2014; Tofteng & Husted, 2011; Wake, 2013)
- Building indigenous identities as well as in establishing the legitimacy of claims to land and heritage ( Levy, 2006)
- Social reproduction: reflecting the shape, values and practices of dominant groups, at the expense of the marginalised ( Dawson, 2018)
- Obscuring postcolonial realities ( Adams, 2017; Im, 2008)
- Critical awareness/changed behaviour concerning health problems among populations and practitioners ( Conquergood, 1988)
- Community empowerment ( Sonn *et al.*, 2015).

There are many points to be made from these results. For example, although Conquergood’s (1988) article discusses performance arts as a means to create awareness of health issues, the article argues above all for the dependency of interventions on the participant population, and to consider the culturally appropriate means of reaching out to the population on their terms, i.e. the social prerequisites for working with and changing conditions for a group of people through art interventions. The article identifies a frequent mistake among health workers working with refugees: blaming disadvantaged groups for their situation. Thus, it is reasonable to claim that the article discusses the social effects *on* art rather than the social effects *of* art.

Several other articles reverse the discussion and ask questions: How do new trends in knowledge dissemination affect museums ( Balloffet *et al.*, 2014)? What is the importance of national context and global hierarchies for arts journalism covering non-western culture/arts ( Janssen *et al.*, 2008)? How are globalising trends towards interculturalism and modernisation mirrored in national/local theatre developments, and with what effects? ( Adams, 2017; Im, 2008)? These are a few examples that made us aware that our method is not sophisticated enough to separate studies of the social effects of art from studies on the social effects *on* art. Perhaps, as in all meaningful activities made by humans, such distinctions are not readily made.

This brings us to the results of the analysis which point to the articles which thematize the negative social effect of art, especially in the sense of being used to silence marginalised voices and experiences ( Dawson, 2018; Edmondson, 2005; Levy, 2006), to evoke negative emotions or ethically problematic assumptions in production and dissemination ( Heddon *et al.*, 2012; Moneta & Rousseau, 2008; Wake, 2013), or to obscure or make light of colonial pasts ( Adams, 2017; Im, 2008). These results both confirm and point to a different kind of negative effect than the theories of Bourdieu (1984); Bourdieu (1984) and answer the tentative question of Belfiore & Bennett (2007) about unquestioned assumptions about art in cultural policy discourse: can the arts be negative? Through this review, we can say that the arts can have negative effects, depending on how they are used. For example, Tofteng & Husted (2011) claim that “theatre-based action research opens up a new way to communicate and make visible knowledge and experiences from below that have difficulties reaching the public agenda or influencing structures of power (abstract).” However, when the analysed play reached the audiences, “One of the scenes was met with massive scepticism” and ended in “heated debate” (p.37) where the audience expressed disbelief in the participants' story of being refused income support and dismissed the experience as “fantasy or pure fiction.” The play was subsequently changed to display an experience the participant never had. Undoubtedly, this must have been a harrowing experience for the participant, and the effects ultimately proved to be artistic, not social. However, the author does not account for this episode as part of the effects of art or artistic choices.

What effects are not studied? In our review of the research front, we find no studies that conceptualise the entertainment or enjoyment aspect of arts and culture as a potential effect and which place importance on the micro, meso, and macro levels, apart from Rickard, Bambrick & Gill (2012) who emphasise “the intrinsic value of music education for enjoyment and learning” (abstract).

This analysis of the research front adds perspectives but also confirms that the outcome to develop skills or to gain knowledge is the most researched, and validated, type of effect found in the data. In what ways this effect can be claimed to be *social* requires a theoretical discussion on the links between education’s micro and macro impact.

## Conclusion: understanding the social impact of the arts through mapping the research field

This study aimed, through bibliographic methods, to define and survey the research area “the social impact of the arts”, to understand the major themes and characteristics of the field, including in what we operationalise as the ‘research front’ in the field of the social impact of the art. As such, we sought to provide insights into categories, patterns, and trends in the field.

Firstly, through identifying relevant databases and search strings, along with using the PIO-model for identifying population, intervention, and outcome in the data to retrieve more relevant hits, we created a data set of over 10,000 articles that constitute the research field “art’s social impact.” Arts and drama education journals were found to be most common in the field, along with a few journals on museum work/theory. The number of publications has risen from 2015 onwards, which indicates that research interest in the arts’ social impact is growing.

Through a co-word analysis, we identified topical clusters within the data, consisting of terms often found co-located with each other in the texts. These were categorised as three overlapping but identifiable themes that, through a close reading of typical articles in each theme, were found to constitute subfields of their own right in terms of epistemologies and methodologies. These were categorised as: 1. social sciences/humanities research on the meaning of arts culture, 2. arts education research on the meaning of arts for learning/skills outcomes, and 3. research on art as a means of health and wellbeing. Through the analysis of these themes, we can understand art’s social impact as relating to health and wellbeing, education and knowledge (or cognitive learning skills), and community and identity. Where the PIO categories are distinguishable, the child in a formal learning context is the most common targeted population, theatre/drama is the most common intervention, and knowledge/skills enhancement is the most common outcome. This reflects the dominance of arts education journal articles in the data.

Moving on to an analysis of the research front, operationalised as the most cited articles in the data set, we again used the PIO model to distinguish research categories and discuss the meaning of art’s social impact in the data. We concluded that the concept of “art,” defined as aesthetical activities, is too narrow to understand the kinds of cultural/artistic interventions/themes explored in the data and needs to be discussed concerning a more general “culture” category that can encompass the actions of institutions. The research front of art’s social impact is about marginalisation issues such as minorities, refugees, and disadvantaged groups. Still, children and young people were found to be the most targeted research population, which confirmed the co-word analysis. Theatre and drama performances (either for or with the target group) are the most common interventions, but we also frequently found articles relating to a more general media/popular culture theme, which relates to the desired outcome in many articles, which is theory/method development. This theme, along with articles aiming to evaluate/assess cultural policy or cultural management tools, added little to the discussion on the arts’ social impact, as they either take the social good of the arts for granted, or aim to add to their research field without theorizing the significance of their results outside theory/methodology development. More direct reading of the articles, with clearly defined exclusion criteria, is needed to investigate further the meaning of art’s social impact in research. 

Regarding outcomes, other themes from the analysis were: fostering ethicality and moral behaviour on audiences and participants, health/wellbeing, community empowerment/identity, and most commonly, skills enhancement and knowledge dissemination/learning. Although this study did not have an evaluative aim, it was sometimes difficult to understand if and how impact has occurred, especially regarding fostering ethicality and moral behaviour among audiences. Another theme discussed in the paper was the investigation of how various social issues impact art and artistic practices, rather than the other way around. Concerning this, the article discusses the negative impacts of the arts in research as a result of analysis, with examples of using the arts to silence marginalised voices, to evoke negative emotions or ethically problematic assumptions in production and dissemination, and to obscure or make light of colonial pasts in artistic production.

In the article, we discuss the meaning of art’s social effects. Often, the research reflects effects on group or individual levels but seldom theorises links between the micro, meso, and macro levels. Beyond the effects of knowledge and skills enhancement as a result of various art interventions, the effects that can be claimed to be social and “positive” in research are: reaching the public agenda, achieving media response (which can be negative), influencing structures of power, building indigenous or community identities with importance for resisting the majority culture, and achieving critical awareness and changed behaviour concerning health problems (in specific circumstances). These categories, along with the arts and culture as a means for knowledge and skills enhancement, are interesting to further investigate in relation to the social value of the arts and may deserve policy attention. Few articles discuss the enjoyment or entertainment aspect of arts and culture.

The methodology of studying the research front has provided us with interesting categories for understanding how art’s social effects are studied in research. Future studies can dive deeper into understanding each category and do a more evaluative reading of claimed results.

## Data availability

All data underlying the results are available as part of the article and no additional source data are required.
