# Mapping research on the social impact of the arts: what characterises the field?

**DOI:** 10.12688/openreseurope.14147.2

**Published:** 2022-08-18

**Authors:** Sofia Lindström Sol, Cia Gustrén, Gustaf Nelhans, Johan Eklund, Jenny Johannisson, Roger Blomgren

**Affiliations:** 1 The Swedish School of Library and Information Science (SSLIS), University of Borås, Borås, Sweden

**Keywords:** artistic intervention, effects of culture, bibliometric analysis, cultural policy, arts and culture

## Abstract

This article explores the broad and undefined research field of *the social impact of the arts*. The effects of art and culture are often used as justification for public funding, but the research on these interventions and their effects is unclear. Using a co-word analysis of over 10,000 articles published between 1990 and 2020, we examined the characteristics of the field as we have operationalised it through our searches. We found that since 2015 this research field has expanded and consists of different epistemologies and methodologies, summarised in largely overlapping subfields belonging to the social sciences, humanities, arts education, and arts and health/therapy. In formal or informal learning settings, studies of theatre/drama as an intervention to enhance skills, well-being, or knowledge among children are most common in our corpus. A study of the research front through the bibliographic coupling of the most cited articles in the corpus confirmed the co-word analysis and revealed new themes that together form the ground for insight into research on the social impact of the arts. This article can therefore inform discussions on the social value of culture and the arts.

## Introduction

The notion that art impacts society beyond its aesthetic value has a long history, dating back to the concept of catharsis proposed by Aristotle ( Belfiore & Bennett, 2007). Since the 1990s, the social effects of the arts in a European context have been increasingly explored by researchers, policymakers, and other stakeholders ( Belfiore, 2002; Belfiore & Bennett, 2010; Gray, 2007), and ‘social impact’ often forms a justification for public funding of the arts ( Belfiore, 2002; Gray, 2007). For example, from 2016 to 2018, the Swedish government funded arts projects in socio-economically marginalised areas to advance democratic ideals and cultural participation, inspired by the Art Council England’s ‘Creative Spaces and People’.

This study is written in the context of the Horizon 2020 EU-funded AMASS Project (Acting on the Margins: Arts as Social Sculpture), which aims to address specific aspects of marginalisation in Europe through arts and creative activities ( AMASS, 2019). The testbeds, comprising various art projects, are situated in the Czech Republic, Finland, Hungary, Italy, Malta, and Portugal, countries with a marginal position, geographically, socially and/or culturally, in the European Union. As an overarching project, AMASS is part of a policy trend to explore the potential of the arts to address social marginalisation in a changing Europe. Within the project, ‘the arts’ primarily refers to the performing and visual arts, and ‘social marginalisation’ primarily refers to individuals, populations, and communities that have not been able to fully exercise their social rights, in terms of, for example, access to educational systems and the labour market.

Thus, from a policy and practitioners’ perspective, the arts are often laden with a positive value, although some research argues that the arts exist only for those who can decipher them: a real impact of the arts can exist only for a small elite of educated people ( Bourdieu, 1984). The mechanisms of impact – either in the form of consumption or production of the arts – on individuals and societies are still unclear ( Belfiore, 2002; Gilmore, 2014), and the research field of “the social impact of the arts” is vaguely defined and therefore difficult to assess.

In this article, we aim to characterise the field through an overview of trends in studies on the social impact of the arts as a broadly defined research area. What, when delineated and defined according to the criteria set within the AMASS project, are the major themes found in this research field? What are the characteristics of the ‘research front’ in the social impact of the arts? With this study, we wish to inform debates on the social effects of the arts and provide cultural policy scholars and artist-researchers with insights into categories, patterns, and trends in the research field of the social impact of the arts. In a previous study ( Gustrén *et al*., 2021), we analysed ‘grey literature’ on the social impact of the arts. This study focuses on peer-reviewed research in English, a selection that is discussed in detail below.

### Surveying a field: a literature review

When surveying a clearly defined field of research, scholars often use a systematic literature review (SLR) to map and assess current knowledge ( Kitchenham, 2004; O’Brien & McGuckin, 2019). In the field of the social impact of the arts, studies such as Daykin *et al*. (2008), Jindal-Snape *et al.* (2018), and Young, Camic & Tishler (2016) provide valuable insight into topics such as the effects of the arts on adolescent health and behaviour, the general academic performance of children, and on cognitive functioning in elderly people with dementia. This study takes a broader look at an unclearly defined field. In this article, we do not perform a strict SLR, rather we employ a more open approach where a vital objective is *to illustrate the potential variety in the totality of different understandings of the social impact of the arts present in the research literature*. The method is inductive, and thus we do not start with preformed hypotheses guided by an established theoretical approach. Our ambition is for our results to serve as a first step in theorising the question of the impact of art on marginalised groups. However, in line with the SLR method, we attempt to analyse available research as thoroughly, fairly, and with as little bias as possible ( Kitchenham, 2004; O’Brien & McGuckin, 2019).

In the following sections, we outline the two-step analysis that we performed. The first step delineated the field. We formulated a review protocol to collect data, which we thematised and visualised using a co-word analysis. To identify the characteristics of the emerging themes, we performed a qualitative analysis by reading selected articles from the corpus. In the second step of the analysis, we performed a bibliographic coupling on the reference lists of the articles in the set to identify the research front. These consisted of works with similar thematic content, defined as the most cited articles in the corpus. This generated a new set of themes which were also qualitatively assessed through a close reading of selected, highly cited articles within the set. We conclude our study by discussing the implications and limitations of our findings and making suggestions for further research.

## Methods

### Delineating the field

We began by conducting a pilot study to identify relevant databases and search strings. The research team performing the pilot study included two university librarians who conducted literature searches in Web of Science, SCOPUS, Art & Architecture Source (EBSCO), and general search engines, by applying a broad set of keywords.

Based on the pilot study results, we concluded that the review should include additional databases covering scholarly publications and should exclude general search engines because they provided irrelevant hits. The keywords were then limited to generate more relevant hits in databases. For the study, the delimitation of the search terms was motivated by themes present in the AMASS project, by defining arts as *mainly* relating to performing and visual arts. Secondly, we define “social impact” as relating to issues of marginalisation explored in the project, such as poverty, minority issues, and forced migration, i.e., in terms of underserved or marginalised communities, populations, or individuals. In relation to these issues of marginalisation, we primarily searched for the effects of interventions aimed at addressing them in some way.

To generate more relevant hits in databases, we limited the keywords within the PIO framework (population-intervention-outcome), inspired by a model used in pilot studies for evidence-based research in nursing ( Arguelles, 2011) ^1^. Based on the results of the pilot study, we developed the following review protocol:

***Time frame*** *:* 1990 – 2019. The time frame was set to make an analysis of development over time possible while allowing only for literature available in the digital format.

***Literature included*:** Peer-reviewed articles in English, available in the digital format.

***Databases*:** Searches were conducted in two general databases, SCOPUS and Web of Science (Arts and Humanities Citation Index), and eight more specialised databases: Art & Architecture Source (accessed from EBSCO); Art Bibliographies Modern (ABM); Arts and Humanities Database; Design and Applied Arts (DAAI); ERIC; International Bibliography of Art (IBA); PsycINFO; and Sociological Abstracts (all accessed from ProQuest).

***Keywords and search strings*:** Within the framework of PIO (population-intervention-outcome), “social exclusion” OR “minorities” OR “marginalised” were set as the main keywords for population (with 12 keywords as subordinated variants), “performing arts” OR “visual arts” were set as main keywords for intervention (with 21 additional keywords as subordinated variants), and “social impact” OR “empowerment” OR “policymaking” OR “evaluation” were set as main keywords for outcome (with 25 keywords as subordinated variants, see Appendix 1 for a complete list of keywords). When conducting the searches in each database, the three sets of keywords were combined by using AND ( Table 1).

**Table 1.** Search terms according to the PIO model.

Population	Intervention	Outcome
*Main search terms:* social exclusion, marginalisation, minorities	*Main search terms:* performing arts, visual arts	*Main search terms:* social impact, empowerment, policy making, evaluation
Indigenous Native Immigrant Migrant Refugee Intercultural* Diversity Underserved Underprivileged Poverty Gender Children Young people	Drama Theatre Theater Museum Performing art Contemporary art Arts education Art intervention Artistic project Community art Socially engaged art Participatory art Arts activism Public art Civic art	Social effects Social change Social outcomes Wellbeing Well being Health Mental health Quality of life Inclusion Citizenship Civic engagement Civic participation Equity Social equality Values Attitudes Tolerance Resilience Empowerment Skill enhancement Evidence Measurement Analysis Assessment Democratic development

The search resulted in 11,764 hits in 10 databases, chosen for their appropriateness for the subject ( Table 2).

**Table 2.** Distribution of identified documents from each source.

Database(s)	N
Arts and architecture Source (EBSCO)	2,374
Arts and Humanities Citation Index (Web of Science)	974
SCOPUS	2,850
Various Proquest databases	5,566
**Total**	**11,764**

The removal of duplicates stemming from the combination of databases using DOI (1404 duplicates) and title (133 additional duplicates) resulted in a total amount of 10,227 unique documents.

Although care was taken in retrieving documents, we identified some quirks in the respective databases during the process. Firstly, although the time frame for the retrieval was bound from 1990 to 2019, some articles published outside these limits were found. Ten articles had no registered year. Sixty documents were published in 2020, and five had a publication year between 1978 and 1988. We did not investigate the reason for this further but surmised that different databases use different ways of identifying the year in the search (i.e., publication year versus submission year, the existence of preprints, and the possibility of metadata errors in the included databases). Because we deemed that the bias introduced by their inclusion would be negligible for the final analysis, these papers were included in our corpus.

### Outlining the field: descriptive analysis

**Figure 1.**
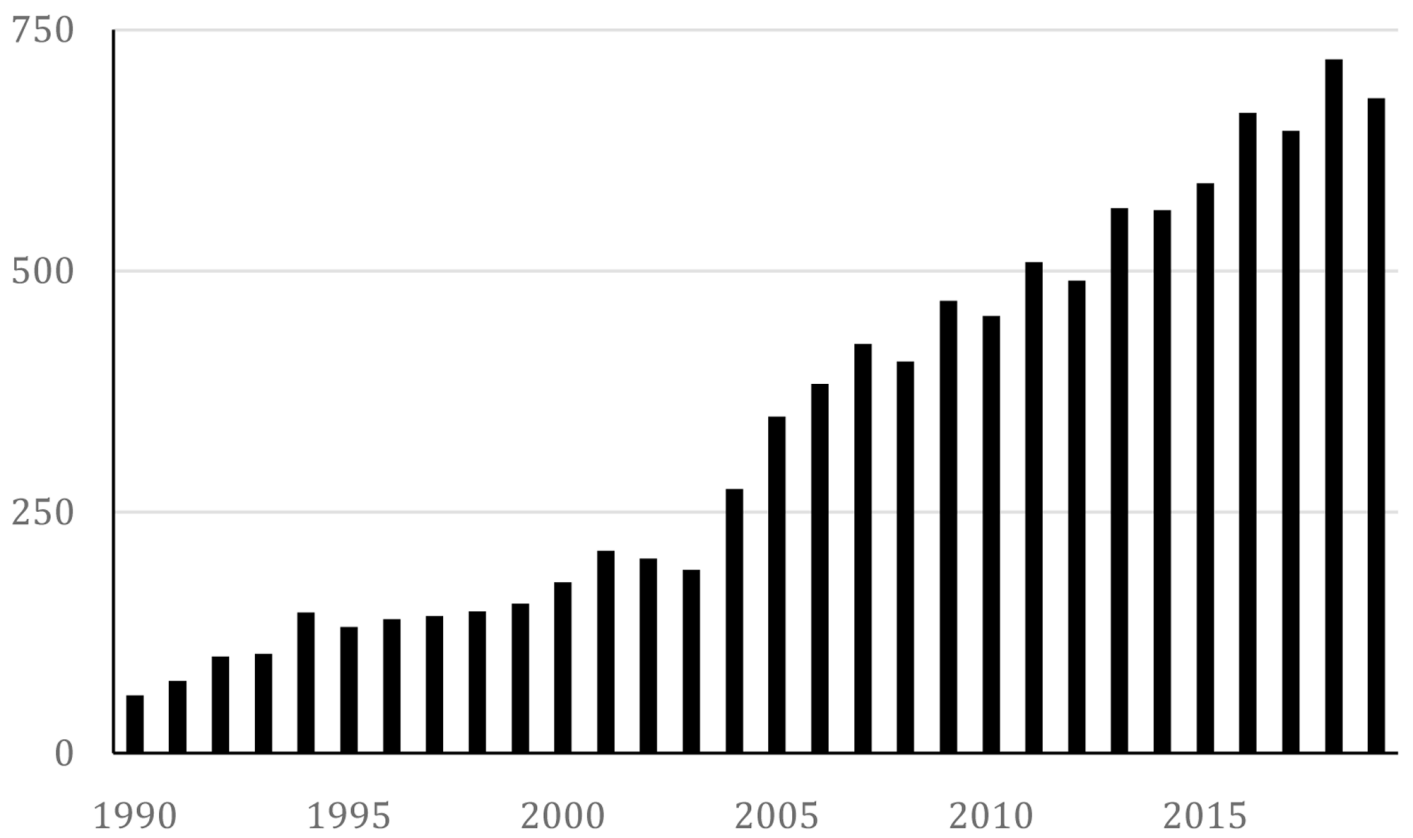
Number of retrieved documents yearly.

The bibliographic approach amounted to a description of the top 20 journals where the articles were published and a count of the number of articles published each year, summarised in Table 3 and Figure 1.

**Table 3.** Top 20 journals in the set.

Publication title	Count of final order
Arts Education Policy Review	210
Art Education	120
Research in Drama Education	119
Visual Arts Research	114
Studies in Art Education	98
Journal of Aesthetic Education	94
Curator	92
Museum International	89
New Theatre Quarterly	87
Youth Theatre Journal	82
International Journal of Art & Design Education	62
Journal of Museum Education	55
Journal of Archaeological Science	54
Theatre Topics	52
Theatre Survey	51
International Journal of Education & the Arts	51
Antiquity	50
Third Text	46
Museum Management & Curatorship	43
International Journal of Education through Art	41

This list illustrates that the discipline of arts education is dominant in the field and that journals dedicated to research on theatre and museums are at the forefront. Among the top ten journals, five are indexed in Web of Science. The other journals are indexed in SCOPUS, except for *Visual Arts Research*, which is not represented in Journal Citation Reports or SCOPUS.

From this figure, we can derive that research interest in the social impact of the arts, interpreted as the number of articles published per year and operationalised through our search strings, have increased since 2005 and tripled in amount to around 600 in 2015. Thus, it is an expanding field, which further motivates the need for mapping trends in the data.

### Analysing emerging themes in the data

**Figure 2.**
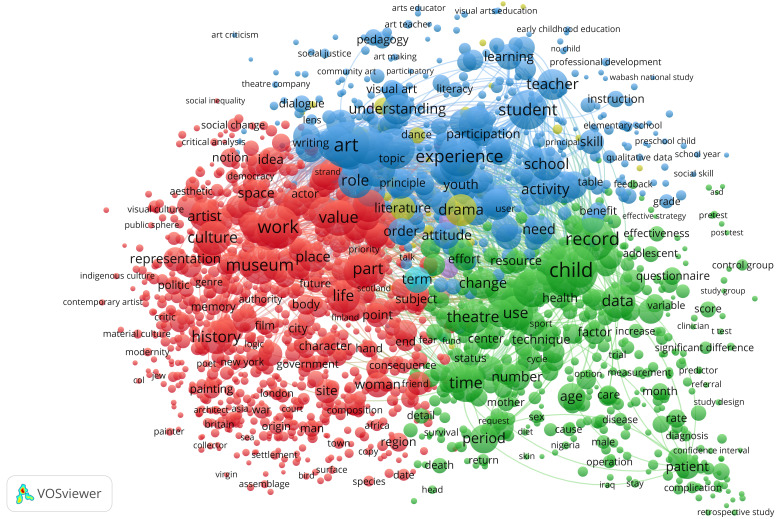
Co-word analysis of terms found in titles and abstracts in the data set. The co-occurrence of 1827 noun phrases found at least 20 times in the data set was used to construct the visualisation. Three main clusters were identified based on the thematic closeness of the terms in the texts.

We performed a text-based content analysis to understand emerging themes in the data, which used the terms found in the titles and abstracts of the collected publications to identify the topical structure of the data. Co-word analysis was used to identify topical clusters within the collected texts, consisting of terms often found co-located in the texts. Using the software package VOSviewer, an analysis of the contents of the titles and abstracts of the articles was performed and visualised as a co-word map. VOSviewer employs a method for “linguistic” parsing (the Apache OpenNLP programming library, Van Eck & Waltman, 2010) that identifies noun phrases, meaning words, or combined terms including nouns or adjectives in front of nouns in a data set. We identified that the co-occurrence of these noun phrases calculated a distance measure based on the co-occurrence of each pair of noun phrases. Lastly, a threshold for inclusion in visualisation was applied, limited to noun phrases occurring at least 20 times in the text (n=1966). In our treatment of the data, we found that generic terms, such as ‘article’, ‘author’, and ‘paper’, obfuscated the results in the visualisation. We therefore performed a manual inspection of the noun phrases and removed these generic terms. The resulting data set used in the analysis consisted of 1,827 terms. The visualisation of the terms used in the articles presented us with three overarching themes, demonstrated in three clusters ( Figure 2).

We named each cluster based on the topic areas related to artistic impact in the graph. Since the co-word analysis is calculated as the statistical relationship between all terms, there is an overlap between the categories. The boundaries between categories are more blurred than the colours indicate. This does not mean that the clusters cannot be distinguished from each other, rather that the terms are gradually more related to each other as they get closer to the boundary. The red and the green clusters are abutting the blue cluster to suggest a correspondence between terms used in articles from all three clusters. For instance, terms like ‘attitude’, ‘order’, ‘change’, ‘effort’, ‘part’, and ‘subject’ link these three clusters together in the overarching themes of culture, learning, and health. An analysis of word occurrences in the cluster resulted in the thematic identification described below.

**The red cluster** is mainly concerned with *issues of culture and society*, such as the value of culture within the geographical context of cities, municipalities, countries, or regions. The terms within the red cluster consider aesthetic culture, i.e., primarily the fine arts, as well as anthropological culture, i.e., a whole way of life ( Williams, 1985). In this context, culture also includes the museum as an institutional space as well as the artwork and artefacts housed in cultural heritage institutions. Although ‘women’ is a term in this cluster, it typically lacks a specified analytical subject.

**The blue cluster** thematises *issues of art and education*. It typically deals with the experience of learning in an institutionalised and formal setting or a more informal community setting. The typical research subject in this cluster is the pupil or, more broadly, the child.

Lastly, **the green cluster** connects *issues of art and wellbeing*. It is primarily focused on health issues in context, with methodologies and techniques for measuring the use of art to improve health and well-being among the subjects of intervention.

The visualisation helped us to analyse how these clusters represent various epistemological and theoretical traditions that overlap but are still disparate enough to be categorised as subfields. Similarly, the bibliographic method allowed us to cast a wide net and work inductively to survey the field. However, it does have certain limitations. Fields with a high number of publications, and that more readily align themselves with the PIO framework, emerged to a higher degree than other fields in our searches.

***Selecting articles for a qualitative analysis of the themes.*** To validate the thematic structure of the field, we performed a close reading of articles representative of each cluster based on the co-occurrence of terms in the visualisation. We used an approach based on the term weighting of the titles and abstracts to identify relevant articles to read. We wrote a script that determined the number of times a specific term was found in each text and a relevance measure that determined how distinctive the specific term is in the corpus. Using the visualisation, we identified specific terms used for selecting relevant articles to read in the qualitative part of the study. Initially, we focused on the ten most common terms per cluster, five relating to the other clusters and five isolated terms in each cluster. The isolated terms were interpreted as those that set the theme apart, while the related themes provided articles that can be argued to belong to overlapping themes. This work resulted in twenty distinctive terms for each of the identified thematic clusters found in the visualisation. We identified fifty articles for each term and used the metadata consisting of title, journal information, and DOI to determine the actual articles for close reading. All in all, one hundred articles were read for the analysis.

Based on the three identifiable clusters generated by co-word analysis, the specific terms we used to identify documents were:

**Culture and society cluster**

Terms related to the other clusters: “ *value,” “part,” “idea,” “subject,” “country.”*

Terms isolated from the other clusters: “ *work,” “culture,” “museum,” “place,” “history.”*

**Art and education cluster**

Terms related to the other clusters: “ *experience,” “concept,” “community,” “activity,” “need.”*

Terms isolated from the other clusters: “ *pedagogy,” “understanding,'' ''student,” “teacher,” “learning.”*

**Art and well-being cluster**

Terms related to the other clusters: “ *record,” “child,” “change,” “theatre,” “time.*”

Terms isolated from the other clusters: “ *patient,” “treatment,” “age,” “data,” “use.”*

Given that the research articles thematised art and culture, we performed a close reading of the articles in dialogue with one another by using three guiding questions in the analysis: How do the articles conceive of the role or effect of the arts? How is this role or effect realised/explored in research? What conclusions do the articles wish to disseminate to the research community? These questions directed our analysis of this material to the overall research questions and aim of the study.

### Results: the arts and society, education, and well-being

Our thematic analysis confirmed that the clusters overlap in themes but are epistemologically distinct. They also vary in terms of methodologies. *The culture and society cluster* represents research that takes a more general outlook on the role of arts and culture in society, in contrast to research with a greater focus on certain populations of interest (such as children and young people and their developmental abilities, behaviours, or attitudes). Typical research themes are related to debating *the (changing) role of the arts* (e.g., Delacruz [2011]; from the isolated term ‘work’). Within the same theme is an exploration of *the changing role of cultural institutions*, such as museums and libraries (e.g., Burgess [2009], within the isolated term ‘history’; Martens [2015], related to the isolated term ‘culture’). Drawing on the concept of ‘site’ as a meaningful place or location, Morris & Cant ([2006]; within ‘place’ as an isolated term) critically examine the relation between art and places through observation of artistic practices. Thus, the isolated terms are found in studies that debate the role of arts and culture in a community context by critically approaching the significance of artistic practices for society rather than focusing on methodology. Articles within the terms ‘culture’, ‘history’, ‘work’, and ‘place’ are also rather broad in scope, indicating less group-oriented or targeted interventions and more interventions directed towards the general public.

Articles with terms that are isolated from the other clusters conceptualise the role of culture and the arts to reflect developments in society and foster shared values for social welfare, often through cultural institutions. These articles tend to offer reflective narratives, as opposed to more traditional scholarly texts in the social sciences where specific theoretical assumptions are set against empirical material and conclusions are drawn (although such research exists). Methodologies are not fitted into the PIO model of experimental research but were philosophically/theoretically oriented, sometimes lacking empirical material. The tendency to include more general social developments as a theme reflects a more humanistic rather than social science or natural science approach. In the art and well-being cluster, the term ‘subject’ primarily refers to children and their development, whereas ‘value’ relates to the isolated term ‘museum’ in the art and society cluster. The term ‘part’ may also relate to the lifecycle (birth, death, etc..) of the art and well-being cluster, in that the health conditions of older people are discussed in relation to their engagement with the arts. Finally, the articles associated with the term ‘country’ link together the clusters in the sense that they are about community art and learning, and a connection to the lifecycle of people in specific geographical places. For example, exploring mural paintings and developing “methods which had the capacity to tell stories of coexistence on the country, in a place where Aboriginal and non-Aboriginal people have been immersed in a cycle of relations for over 230 years” ( Harrison *et al*., 2016, p. 1330; within the related term ‘country’).

Research in the *art and education cluster* is characterised by a strong belief in the significance of the arts and their impact, mostly as a learning tool, and often details results of this approach in different contexts. For example, how theatre pedagogy can be used in intercultural education research ([ Frimberger, 2016]; ‘pedagogy’ as an isolated term), how young people are trained to theatrically explore their identities and the identities of others ( Reisberg *et al.*, 2006; Tuisku, 2015), or how art education can help students acquire knowledge and apply concepts at different levels of cognitive development ([ Ho & Lin, 2015; Lekue, 2015]; ‘understanding’ as an isolated term). The articles we studied indicated that the relationship between teacher and student is more of an agreement than one characterised by a power imbalance. The teacher is not so much in focus as the student-actor or spectator ([ Stillman & Beltramo, 2019]; ‘teacher’ as an isolated term). Hence, pedagogy has more in common with the practice of creating artwork, although studied from the perspective of art educators and the de-centring of art education. The term ‘activity’ thus relates to the art and well-being cluster of the development and health of children. Knowledge development through art is conceived of the visual component of learning and has connections to self-expression. Understanding, literacy, and psychology are about the psychological and sociocultural development of children regarding their aesthetic and conceptual abilities to articulate or represent knowledge. The related terms, such as ‘activity’, are close to the art and well-being cluster and take an art-therapeutic approach. One important conclusion is that the concept of ‘community’ in this cluster is participation oriented. Research associated with this term aims to show the value of art education for individual community members and communities at large. Articles within the arts and education cluster do not always explore formal learning institutions or situations. Bailey *et al.* ([2019]; within ‘need’ as a related term) addresses the future needs for qualitative, accessible, and appropriate housing for the elderly, where the role of art, in this case serves to open channels of communication. Furneaux-Blick ([2019]; ‘activity’ as a related term) conceptualises art therapy as meeting clients on their terms and addressing the power imbalance inherent in working with children with (in)visible disabilities. Articles in this cluster can thus promote the significance of art education by bringing it out of the classroom and offering it openly to benefit the community at large ([ Ortiz & Chung, 2011]; ‘community’ as a related term). The related terms of the art and education cluster are thus clearly related to the art and well-being cluster and their subject matter specifically explores issues related to the elderly and students with (in)visible disabilities. The isolated terms are more student-oriented and situated in a formal learning context, in contrast to related terms that form a bridge back to the art and society cluster and its focus on community welfare.

Research typical for *the art and well-being cluster* both in terms of belonging to terms isolated from and related to the other clusters, encompasses topics related to the understanding of how various aspects of art in various settings and contexts can impact a set of specific populations. These include nurses, children in paediatric care, women from a specific community, or school children. Research in this cluster is exemplified by the need to articulate the aims and objectives of cultural provision and generate ‘evidence’, such as the impact of youth theatre on non-arts audiences ([ Hughes & Wilson, 2004]; within ‘theatre’ as a related term). Findings in this cluster were discussed in tandem with theory, especially in research belonging to the isolated terms (for example, understandings of youth transitions theory and Piaget’s (1997[1929]) developmental stages of the child). Research belonging to the isolated terms was found to be oriented towards educational professionals, such as insights into developing art therapy methods for autistic children ([ Cao *et al*., 2013]; within the isolated term ‘treatment’). Thus, this cluster represents an experimental theme focused on methodologies to capture the effects of arts interventions. The more isolated the term, the more this focus on methods related to a specific medical field, but the cluster is not restricted to studies of the impact of the arts in medical research. As studies involving children are common in this cluster, ethical issues regarding interventions for children demand rigorous methodologies for conducting and capturing the effects of the arts and culture.

There is a typical research design in this cluster of combining a quantitative and qualitative approach with participatory involvement of the population being investigated (conceived of as “peer researchers”), such as questionnaires, qualitative interviews, and participatory creative research workshops. Impact is thus an essential concept for research representing this cluster, for example, the impact of art on improved health and satisfaction outcomes in paediatric patients ([ Nanda *et al.*, 2009]; ‘age’ as an isolated term). Research in this cluster report on mixed methods, such as surveys and recorded qualitative comments for finding significant differences in art preferences across the different age groups, ( Nanda *et al.*, 2009) or using both questionnaires and focus group studies to approach film as a platform to engage, evoke, and develop understanding of the patient experience among student nurses ([ Ogston-Tuck *et al.*, 2016]; ‘patient’ as an isolated term).

Typically, the articles in the related terms of the *art and well-being cluster* showed a topical diversity, compared to the isolated terms related to a medical research tradition. For example, research in this cluster within the related term ‘change’ involved developing tools for understanding how the short-term effect of visiting a science centre exhibition in Finland influenced the knowledge and attitude of young people toward climate change ([ Gorr, 2014]; with the results of the study indicating that the exhibition experience did not support comprehensive attitude change). There were also studies aimed at providing researchers and practitioners who work with children with a model for creating guided-play activities in which children experience a range of emotions. Thus, according to the researchers, they develop social and emotional skills ([ Goldstein, 2018]; ‘time’ as a related term). Other research was more user-oriented, focusing on the participatory element, such as Brahma, Pavarala, & Belavadi, ([2019]; within the related term ‘record’) who studied the use of theatre targeting regressive social norms relating to gender inequality, such as domestic violence, child marriage, women’s and children's health issues.

In summation, these themes present the social impact of the arts as it relates to health and wellbeing, education, and knowledge (or cognitive learning skills), and – somewhat less identifiable – community and identity. Where the PIO categories are distinguishable, children were identified as the primary population under study, and often in formal learning contexts. Theatre and drama are the most common interventions, and knowledge/skills enhancement is the most common outcome. These clusters overlapped where articles included a methodological exploration in understanding how the arts impact either individuals, groups, or more generalised populations. For example, SmithBattle ([2012]; ‘student’ as an isolated term) studies the pedagogy of student-created dramatic performances to promote reflection on sexuality and health care experiences. The role of theatre is understood to “foster empathy and convey the complexity of clinical situations from the vantage point of patients and clinicians” ( SmithBattle, 2012, p. 591; c.f. Feagan & Rossiter, 2011). Articles within the related terms in the *arts and society cluster* contrast with the isolated terms through a more apparent presence of methodological interest or exploration. For instance, they are concerned with patterns of and reasons for engaging in the arts among older people (55+) and barriers to such engagement ([ Keaney & Oskala, 2007]; within the related term ‘part’). In contrast, related terms found in the *arts and society cluster* have an educational focus, such that there is a connection to the *art and education cluster* and its concern with the relation between art and learning. Likewise, Chung & Ro ([2004]; within the related term ‘subject’) studied the effects of problem-solving instruction in practical arts education on the creativity and self-efficacy of children.

Articles within the related terms are not always easily identified to explore the impact of the arts on more generalised populations. For example, Woodward & Ellison ([2010]; ‘experience’ as a related term in the arts and education cluster) examines the transformative potential of subject-object relationships in the context of art. DeBettignies & Goldstein (2020) investigate the role or of improvised drama in improving the self-concept of children as a means of improving their chances to succeed in school (‘concept’ as a related term; and here it specifically refers to self-concept, a multifaceted construct of cognitive and physical abilities and social functioning, status, and recognition).

As our next focus of analysis will show, in our corpus there is also an interest in how societal changes impact culture and the arts. For example, Earle ([2013]; within ‘value’ as a related term in the *culture and society cluster*) investigates how the legitimacy crisis western museums have faced since the end of the 20 ^th^ century has affected their standing as purveyors of cultural education.

## Mapping the research front

In the study of bibliometrics, a research front ( Persson, 1994) is developed by exploring the content among articles with similar references. This builds on the idea that articles that cite similar references carry the “symbolic content” ( Small, 1978) of the cited works. Understanding the research front is sometimes used to determine the ‘best’ research. However, we were interested in understanding emerging themes in such a set of articles to map how a field is formed through related content.

We employed a bibliometric method based on the bibliographic coupling of articles, as quantified by their shared references, to generate a relevant set for our close reading. We therefore reduced the heterogeneous set of articles to thematic groups based on their similarity in content. Using the VOSviewer software, (see van Eck & Waltman, 2010) we analysed a subset of 974 articles ( *W*). We used part of the set of initially identified articles ( *A*), obtained from Web of Science, to produce another cluster analysis of the documents to categorise them into thematic parts. In this instance, the relation among individual articles (identified as ‘author’, ‘year’ in the graph) was based on shared citations in their respective reference lists.

**Figure 3.**
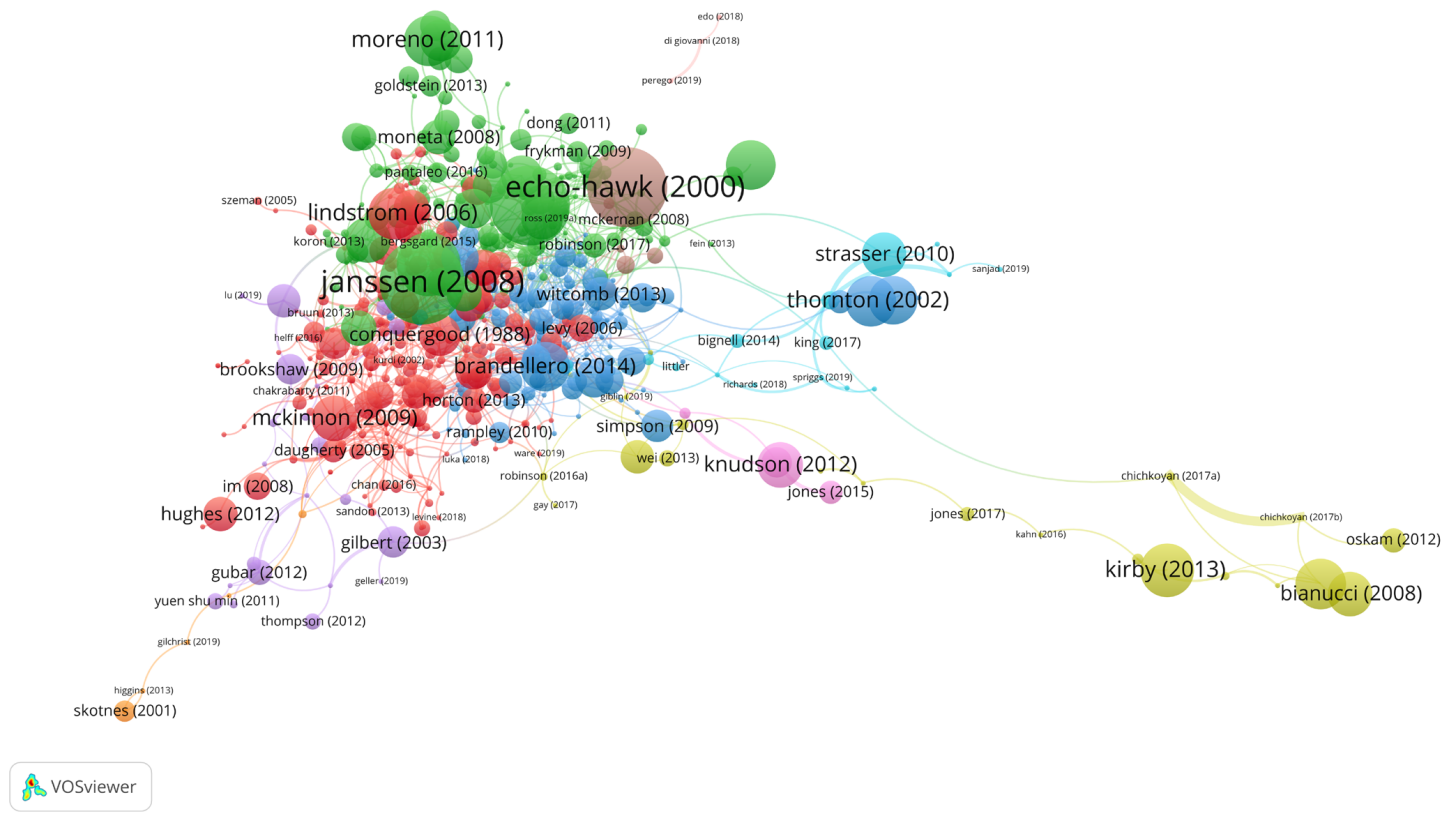
Bibliographic coupling of articles as quantified by their shared references.

The following figure ( Figure 3) visualises the mapping of the most cited articles of nine clusters. This overview illustrates how themes of research can develop as authors connect their work to that of other researchers. In the following paragraphs, we will discuss these themes and their relevance for the analysis of the research front.

### About the clusters

After forming an overview of the nine different clusters of the bibliographic coupling visualisation above, we considered the titles of articles and their thematic content. In doing so, we found that the red and green clusters were most relevant for a qualitative analysis of the research front regarding the art’s social impact. Based on the criteria explained below, we did not examine all the clusters in this dataset, partly to help us define a set of articles for analysis that was manageable within the set time frame of writing (two researchers, Lindström Sol and Gustrén, were responsible for the close reading of the articles).

For example, although the blue cluster (n=99) contained a few interesting contributions regarding the work of cultural institutions, we found several instances in this cluster thematising analysis of objects (e.g., metallurgy, jewellery). Since this was not within our chosen scope, we excluded this cluster from further analysis.

Moreover, the yellow (n=23), orange (n=6), and brown (n=4) clusters mainly contained works on topics like archaeology, biology, palaeontology, and other domains within the natural sciences. They also included humanistic works covering topics in history, prehistory, and antiquity. Ethnographic approaches to the ancient world and its oral traditions were a recurrent feature in these clusters. For instance, Echo-Hawke (2000) was a highly cited article in the orange cluster, whose title and abstract indicate that the article is tracing oral traditions dating back to ancient history. Although interesting, we did not deem it relevant enough for a study on the social impact of the arts from a contemporary perspective.

We made the same determination for the purple cluster (n=16), mainly investigating topics in childhood, nursery, play, and similar features. The turquoise cluster (n=6) explored Elizabethan theatre, and Shakespeare in particular and we could identify no explicit indications of any social effects. Finally, the pink cluster (n=3) covered various forms of audio description or studies of sound in the context of theatre and museum exhibits. These articles were too few and lacked citations, which made it difficult to identify a potential research front.

Initially when developing our corpus, we cast a wide net and sifted for relevant content rather than starting by choosing relevant articles and scrutinising their reference lists individually. This resulted in the development of clusters that we deemed were not relevant to our specific focus of marginalisation. Our method requires manual filtrations, but also has the advantage of excluding reader bias from the selection. Instead, articles with similar reference lists, thus sharing a topical focus, were found close to each other during our analysis. In conclusion, to analyse and define the research front of the social impact of the arts, we performed a close reading of the top-cited articles (determined as those with ten or more citations) in the remaining clusters. We wish to emphasize that literature that was excluded from the corpus based on our selection criteria could still have relevance to the topic area ( Table 4).

**Table 4.** Articles and no. of citations in the analysed clusters.

Red cluster (n=21 of 275)	C (>10)	Green cluster (n=23 of 130)	C (>10)
Lindstrom, 2006	*40*	Janssen *et al.*, 2008	*107*
McKinnon, 2009	*31*	Ash, 2003	*94*
Conquergood, 1988	*25*	Neelands, 2009	*59*
Vannini & Waskul, 2006	*21*	Moreno *et al.*, 2011	*38*
Campbell, 2008	*20*	José del barrio *et al.*, 2009	*36*
Edmondson, 2005	*20*	Peppler, 2010	*31*
Gregory, 2003	*19*	Day, 2002	*26*
Dawson, 2018	*19*	Leonard, 2010	*26*
Hughes, 2012	*18*	Rickard *et al.*, 2012	*26*
Heddon *et al.*, 2012	*15*	Göncü & Perone, 2005	*23*
Henriksen & Frøyland, 2000	*14*	Adams, 2017	*19*
Miller, 2008	*13*	Balloffet *et al.*, 2014	*18*
Lum & Dairianathan, 2014	*12*	Moneta & Rousseau, 2008	*18*
Tofteng & Husted, 2011	*12*	Schuele & Lederman, 2004	*14*
Wake, 2013	*12*	Lauring *et al.*, 2016	*13*
Bernard, 2014	*11*	Eerola & Eerola, 2014	*12*
Im, 2008	*11*	Gibson, 2003	*12*
Levy, 2006	*11*	Greenfader *et al.*, 2015	*12*
Page *et al.*, 2006	*11*	Sapiro, 2015	*12*
Taylor, 2013	*11*	Hsiao, 2010	*10*
Horton & Berlo, 2013	*10*	Octobre, 1999	*10*
		Sonn *et al.*, 2015	*10*
		Wheeler, 2003	*10*

### Analysing the research front: topical themes

Since abstracts and articles in the two remaining clusters did not provide sufficient information about population, intervention, outcome, we did not deem them disparate enough for a meaningful separation of topical analysis, with one exception. Although largely overlapping, the red cluster tends to thematise informal learning settings such as theatre/drama as an intervention. In contrast, the green cluster tends to thematise formal educational settings such as schools and museums.

In our reading, we used the PIO model, i.e., defining population, intervention, and outcomes in the articles, to form a basis on which we can discuss the understanding of the social impact of the arts. By reading the titles and abstracts of the most cited articles in the chosen clusters (n=44), we excluded two articles on the following grounds: the article was not written in English ( Octobre, 1999), and the article did not consider culture and the arts as a theme ( McKinnon, 2009), instead of considering performing vulnerability in the courtroom. This demonstrates that our method requires further refinement to differentiate between figurative uses of ‘performance’. Overall, we analysed 42 articles to understand the scope of the research front of the social impact of the arts.

## Results: an overview of most common populations, interventions, and outcomes in the research front

The included articles were analysed through a close reading to outline the population, intervention, and outcomes, both intended and observed. This presented us with a few interpretational difficulties since most articles were not written within the PIO framework. To identify population, we set out to understand the effects of arts and culture on something or someone, and this something or someone was interpreted as the population. Instead of a defined group of people as the population, it could be more abstract concepts such as arts journalism, political developments and/or the perception of a country or region through art. Similarly, as we will discuss in more depth below, this could include the effects of political and geographical developments on art.

When identifying interventions, we struggled, for example, with the question of what is to be understood as an intervention in studies of theatre plays - the analysis of the play or the actual play? In some of the articles, authors were not part of an investigation of an artistic intervention, rather they engaged in an analysis of cultural products intended to theorise about their significance in various ways. Based on AMASS’ focus on artistic and cultural interventions, we chose to define intervention as the observed artistic/cultural product/process. The emphasis of our analysis was on the observed rather than intended outcomes of these processes.

Interventions provided more straightforward points of analysis. Below we discuss the several categories of observed outcomes we developed to make up the research front of the social impact of the arts.

### Populations

The results of our analysis illustrate that the most common population is children and young people in formal learning settings, and theatre/drama is one of the most common interventions. This confirms that the research front follows the theme of the co-word analysis, where these categories were also common in the overall data. The second category, unspecific or broadly defined populations, related largely to the potential of cultural institutions, such as museums, to act as educational settings. Thus, we conclude that parts of the research front on the social impact of the arts (as defined by our search methods) connect the importance of social impact to the relevance of cultural institutions. Groups experiencing marginalisation, such as minorities and refugees, are not the most prominent populations investigated in our corpus ( Table 5).

**Table 5.** Themes belonging to population in the data analysed.

Populations	Empirical instances
**Children and young people in formal and** **informal learning settings** **(15 articles)**	Children without earlier music training ( Moreno *et al.*, 2011) Primary school child, musical learner? ( Lum & Dairianathan, 2014) Young people/underprivileged youth ( Lindström, 2006; Neelands, 2009; Peppler, 2010) Pupils, children 3, 6, and 12 years old ( Eerola & Eerola, 2014) English learners in the primary grades ( Greenfader *et al.*, 2015) Teacher students ( Page *et al.*, 2006; Gibson, 2003) Art naïve uni students ( Lauring *et al.*, 2016) Primary and secondary school students ( Rickard *et al.*, 2012; Day, 2002) 20 children aged 4–5 ( Hsiao, 2010) Former child soldiers of post-war Northern Uganda ( Edmondson, 2005) Young audiences ( Balloffet *et al.*, 2014)
**Unspecific/broadly defined populations** **(10 articles)**	Individuals ( Vannini & Waskul, 2006) Diverse communities ( Campbell, 2008) The audience participant ( Gregory, 2003; Heddon *et al.*, 2012; Henriksen & Frøyland, 2000) Interpretative communities ( Miller, 2008) Arts journalism ( Janssen *et al.*, 2008) Families ( Ash, 2003) Visitors ( Leonard, 2010) Adults ( Göncü & Perone, 2005)
**Professionals within the culture sector** **(5 articles)**	Regional heritage institutions ( José del barrio *et al.*, 2009) Performers and directors ( Taylor, 2013) Publishers and translators ( Sapiro, 2015) Musicians ( Schuele & Lederman, 2004) Artists and venue providers ( Wheeler, 2003)
**Political geographies** **(4 articles)**	The Israel-Palestine conflict ( Bernard, 2014) Post-colonial Korean society ( Im, 2008) The nuclear in post-war Britain ( Hughes, 2012) Post-colonial Uzbekistan ( Adams, 2017)
**Indigenous, minority people** **(4 articles)**	Indigenous artists, Native North American ( Horton & Berlo, 2013) Sami people ( Levy, 2006) Aboriginal and non-indigenous children, young people, adults in four rural towns ( Sonn *et al.*, 2015) Participants from low-income, minority ethnic backgrounds ( Dawson, 2018)
**Immigrants and refugees** **(3 articles)**	Hmong refugees ( Conquergood, 1988) Asylum seekers ( Wake, 2013) Immigrant adolescents with behavioral difficulties ( Moneta & Rousseau, 2008)
**Disadvantaged (1 article)**	Unemployed people ( Tofteng & Husted, 2011)

### Interventions

The most common interventions confirm the co-word analysis where theatre and drama, in formal and informal educational settings, dominate the research front. Music and visual arts are also common, along with discussions of museum activities. The category ‘media and popular culture’ relates to the outcome category ‘theory/method development’, which will be elaborated on below ( Table 6).

**Table 6.** Themes belonging to intervention in the data analysed.

Interventions	Empirical instances
**Art education/formal learning settings** **(12 articles)**	Student portfolios ( Lindström, 2006) Music/visual arts education ( Lum & Dairianathan, 2014) Drama in schools ( Neelands, 2009) Effect of music training ( Moreno *et al.*, 2011) Use of new technologies for communication as part of arts curriculum ( Peppler, 2010) Forum theatre workshop in school ( Day, 2002) School-based music and visual arts instruction ( Rickard *et al.*, 2012) School based drama intervention ( Moneta & Rousseau, 2008) Extended music curricular class ( Eerola & Eerola, 2014) Attitudes to art and art education ( Gibson, 2003) Drama and creative movement intervention among students ( Greenfader *et al.*, 2015) Art-making activities among kindergarten children ( Hsiao, 2010)
**Theatre** **(11 articles)**	Health theatre ( Conquergood, 1988) Shared memories through drama ( Campbell, 2008) Drama as arts therapy ( Edmondson, 2005) One-to-one theatre performance dialogue and collaboration ( Heddon *et al.*, 2012) Theatre-based action research ( Tofteng & Husted, 2011) Verbatim or testimonial theatre ( Wake, 2013) Political theatre ( Bernard, 2014) Intercultural theatre in Hamlet ( Im, 2008) Verbatim theatre ( Taylor, 2013) Adult improvisation based on child play ( Göncü & Perone, 2005) European style theatre ( Adams, 2017)
**Media and popular culture** **(7 articles)**	Life-from-space theory in popular culture ( Gregory, 2003) Science communication ( Dawson, 2018) Popular imagery and culture of the nuclear ( Hughes, 2012) Digital gameplay ( Miller, 2008) Analysis of international arts and culture coverage ( Janssen *et al.*, 2008) Dissemination of French literature ( Sapiro, 2015) Institutionalization of performance art ( Wheeler, 2003)
**Museum activities** **(6 articles)**	Museums for scientific literacy ( Henriksen & Frøyland, 2000) Exhibition analysis of the representation of Sami identity ( Levy, 2006) Families making sense of science content in museums ( Ash, 2003) Techniques for efficiency evaluation in museum management ( José del Barrio *et al.*, 2009) Exhibitions on popular music ( Leonard, 2010) Edutainment within the museum sector ( Balloffet *et al.*, 2014)
**Visual arts** **(4 articles)**	Attitudes to contemporary, socially orientated art ( Page *et al.*, 2006) Contemporary arts discourse on indigenous understandings ( Horton & Berlo, 2013) Art appreciation ( Lauring *et al.*, 2016) Photography and photo elicitation ( Sonn *et al.*, 2015)
**Music** **(2 articles)**	Aesthetic experience of life through music ( Vannini & Waskul, 2006), Review on occupational disorders of instrumental musicians ( Schuele & Lederman, 2004)

### Outcomes

The most common theme regarding outcomes can be argued to belong to an internal, academic debate, furthering conceptual and theoretical knowledge on the social impact of the arts. The prominent themes of skills enhancement and knowledge dissemination/learning mirror the co-word analysis result, where the social impact of the arts is conceptually related to learning outcomes ( Table 7).

**Table 7.** Themes belonging to outcomes in the data analysed.

Category	Author/year of publication	Intended outcome/aim	Observed outcome(s)
**Theory/method** **development** **(8 articles)**	Gregory, 2003	Assessing a scientific theory’s popularisation via popular press, museums, etc.	The paper argues for science communication studies to look beyond traditional categories to embrace the wide variety of media and genres that contribute to the construction of science in the public.
	Vannini & Waskul, 2006	In this article, the authors explore music as a metaphor for life.	From within the metaphor of life as music, the authors conceptualize beauty as the diverse rhythms, melodies, and harmonies contributing to the constitution of both subjectivity and intersubjectivity.
	Miller, 2008	This article investigates the *Grand Theft Auto* video game series to demonstrate the potential of a folkloristic, ethnographic approach for the analysis of digital games.	This case study suggests that digital gameplay should be regarded as a form of performance practice with the capacity to invoke traditional folkloric genres and engender new traditions.
	Campbell, 2008	This article discusses artistic projects concerning a sharing of the past. It includes theories of memory work, memory sharing as a cultural practise.	The authors states that “diverse audience engagement with political and artistic projects of sharing the past might help fortify the symbolic resources that those who are politically marginalized need to shape or maintain memory” (p. 41).
	Neelands, 2009	This article discusses the ensemble-based approach of drama in schools. It contrasts the pro-social emphasis in the ensemble model with a pro-technical emphasis.	Using ideas drawn from McGrath and Castoriadis, the author claim that the ensemble approach provides young people with a model of democratic living.
	Hughes, 2012	This article studies the effects of cultural interpretations of a phenomenon (nuclear culture) on popular imagery.	The author warns of risk of homogeneous storytelling, and a reduction of pluralistic histories.
	Horton & Berlo, 2013	This study is an analysis of why it has been difficult for the ‘new materialisms’ to incorporate indigenous intellectual traditions into discussions of non-human agency, focusing on contemporary arts discourse.	The authors states that indigenous artists’ understandings of material have an acute awareness of the contemporary, global challenges of cohabitation.
	Taylor, 2013	This article analyses how affective listening is used to develop performances in Alecky Blythe's verbatim theatre.	The article argues that voice in *London Road* both claims and defers authenticity and authority, since voice signifies presence and embodied identity but the reworking of speech into song signals the absence of the real.
**Skills enhancement** **(7 articles)**	Lindstrom, 2006	Can the arts be assessed and taught? This article uses a study of portfolios as methods for art skills enhancement among young people.	The author concludes that the notion that assessments of learning outcomes must be either limited to superficial knowledge or completely arbitrary is a misconception. Improved visual design and artistic skills can be accomplished in certain circumstances.
	Moreno *et al.*, 2011	The article studies the effect of 20 days of music or visual arts training on children’s pre-literacy skills.	The authors found that the children’s “ability to map unfamiliar symbols to known words improved significantly from pretest to posttest” (p. 170). The effects on the music group were stronger.
	Greenfader *et al.*, 2015	The article studies the effect of a performing arts program on the oral language skills of young English learners.	The authors found that the treatment group ( *N* = 902) outperformed controls ( *N* = 4,338) on speaking assessments. Effects were strongest on English learners with the most limited English-speaking abilities.
	Hsiao, 2010	The article investigates methods of enhancing kindergarteners' artistic creative thinking and expressive drawing through appreciation of picture books.	The authors found significant and positive change in reading and drawing behaviours of children when at home. “The results also showed that the collage series of picture books had more impact on children than did other picture books in terms of teaching efficacy by picture book appreciation” (p.143).
	Eerola & Eerola, 2014	The article explores if music education can create social benefits in the school environment (general satisfaction about the school and a sense of achievement and opportunity for students).	The authors found that extended music education enhanced the quality of school life and had a positive effect on the social aspects of schooling.
	Rickard *et al.*, 2012	The article studies the impact of an increase in school- based music training on a range of cognitive and psychosocial measures for 10 to 13-year-olds in two independent studies.	No convincing benefits of school music classes were apparent in the studies. The authors emphasise the intrinsic value of music education for enjoyment and learning.
	Moneta & Rousseau, 2008	The article explores the emotional regulation (ER) capabilities through drama intervention in immigrant adolescents with behavioural difficulties.	Some impairment in emotional expression and emotional regulation in this study sample. “In general, the drama process seemed to help emotional expression and awareness and to foster a transformation of emotive processes in the sense of a ‘collective ER’” (p.329).
**Knowledge** **dissemination/learning** **(7 articles)**	Henriksen & Frøyland, 2000	The article explores “museums’ potential to contribute relevant and usable information in a practical, science- related matter to a local community” (p. 395).	The authors found sceptical attitudes among families and museum professionals towards the role of museums for scientific literacy. This does not threaten the relevance of museums.
	Ash, 2003	The article studies the effects of museums and museum pedagogy on families' scientific sense-making and learning.	The authors argues that themes in knowledge arise from both the family members and the museum exhibit.
	Peppler, 2010	The article studies the effects of the contribution of media arts education in informal settings in learning outcomes among underprivileged youth.	The author argues that through using new types of software, young people can engage with technology in ways that encourages active learning.
	Tofteng & Husted, 2011	The articles studies how drama can empower action research processes in the field of unemployment. The article also discusses the reactions of the audience, “to use the plays to show others how life is outside the labour market.” (p. 37).	The authors argue that theatre-based action research opens a new way to communicate and make visible knowledge and experiences from below that have difficulties reaching the public agenda or influencing structures of power.
	Levy, 2006	The article examines the variable representation of the prehistory of the indigenous Saami peoples in several Nordic museums.	The author found that the presentation of the prehistory of indigenous Saami peoples differs significantly between majority community museums and those run by Saami communities. “The national and regional museums diminish or even deny a Saami role in the antiquity of the nation. In contrast, the Saami institutions grant the Saami the same ancientness as the other Nordic populations.” (p 143).
	Dawson, 2018	The article explores science communication (through popular media and cultural institutions) from the perspective of participants from low-income, minority ethnic backgrounds.	The author is critical towards the status quo of science communication and claims that “Social reproduction in science communication constructs a narrow public that reflects the shape, values and practices of dominant groups, at the expense of the marginalised” (p.772).
	Leonard, 2010	This article studies the effect of museum and museum pedagogy on audiences' sense-making and learning around popular music.	The authors argue that the exhibition “moved beyond the expected to represent a greater diversity of music genres, sounds, performers and dimensions. Value judgements always inform which exhibition ideas are given the green light and shape the way stories are told within displays” (p. 180).
**The impacts of the** **social on art/culture** **(7 articles)**	Lauring *et al*., 2016	This article studies the impact of social and monetary contextual information on liking ratings of art.	The authors found that paintings with high monetary primes or with high ratings by peers and art experts led to higher participant liking ratings. In contrast, paintings with a low rating by the low- education/income social group led to higher liking ratings by participants. These results provide empirical support for the social distinction behaviour theory.
	Im, 2008	This essay examines the implication of interculturalism in New Asia and ultimately the relationship between West and East, focusing on Lee Yountaek's production of *Hamlet* that premiered in Seoul in 1996.	The author argues that Lee Yountaek's adaptation of *Hamlet* reflects the impasse of contemporary Korean society, whose postcolonial reality is obscured by an optimistic idea of interculturalism.
	Balloffet *et al*., 2014	This article examines the concept of "edutainment" within the museum sector.	The authors conclude that edutainment presents both opportunities and risks: attracting new audiences, particularly young people, but also the “Disneyfication” of cultural institutions.
	Janssen *et al*., 2008	The article studies the importance of national context and global hierarchies for arts journalism and the coverage of non-western culture/arts.	The authors found that international coverage remains concentrated on a few countries, of which the United States has become the most prominent. Although the global diversity of coverage has increased, non-Western countries are still underrepresented.
	Adams, 2017	The article examines the cultural change in Uzbekistan through the evolution of European-style theatre during the twentieth century.	The author argues that “the dominance of the European-style theatre means that other ways of being, thinking, and experiencing are marginalized” (p. 354).
	Sapiro, 2015	This article is an empirical study of the circulation of French literature in the United States in the era of globalization.	The author concludes that French literature has “lost its visibility on the American book market” due to the globalization of publishing houses and the transnational literary field (s. 341), and that small publishers have taken over the role of producing symbolic capital.
	Wheeler, 2003	“This article discusses the role of meaning in the institutionalization of performance art between 1970 and 2000” (p. 491).	The author argues that the field has progressed, contradictorily, towards both popular entertainment and avant-garde art, and that artists were committed to “the integration of art with society and the subsequent interest in exposing inequitable social structures” (p. 507).
**Fostering ethical** **and moral behaviour** **in audiences and** **participants** **(5 articles)**	Bernard, 2014	This article discusses plays thematizing the Israel/ Palestine conflict, examining controversies these plays engendered, and “the effort to generate empathy and humanitarian feeling” (…) “and persuade a viewer to affiliate with a particular struggle or set of beliefs (p. 163).	The author claims that these plays “suggest a new receptiveness to Palestinian points of view” (p. 172) but the discussion on emotional effects of audiences is speculative.
	Day, 2002	This study investigates the experiences of, and interactions between, participants of a Forum theatre workshop, which addressed the issue of the refugee child at school.	Findings revealed that the workshop was highly relevant to the students, reflecting moral dilemmas which they faced in their everyday lives, as they encountered refugee students at school. This interactive workshop gave them the opportunity to try out moral behaviour, which could potentially be applied to real-life situations.
	Wake, 2013	This is a study of audience and media reception and the political effects of an Australian play about asylum seekers.	The author hold a discussion on the potential for pain and exploitation in testimonial theatre. “(...) [W]hile *Through the Wire* was ethically problematic, it was also politically efficacious” (p. 117), as “The play was highly visible in the mainstream media. The realist aesthetic facilitated identification with asylum seekers.” (ibid.)
	Heddon *et al.,* 2012	This is a study of effects of one-on-one theatre performance, dialogue, and collaboration on those participating.	The authors discuss a risk of the intervention: the experiential performance proffers hierarchies of experience, invoking the notion of an ideal audience-participant. However, there is also a potential to produce more intimate connections in shared experiences.
**Assessment/** **evaluation** **(5 articles)**	Page *et al.,* 2006	The article studies how socially orientated contemporary art is making a success in the wider global context, but is omitted as practice in many schools, although the UK government recommends art as part of curriculum. How can this be rectified?	“Conclusions have been tentatively drawn about how the curriculum may be better served by the use of contemporary art, as well as the means by which new learning methods may be facilitated” (p. 146).
	Lum & Dairianathan, 2014	This article seeks to assess and evaluate the matching of policy and practice, ambition to include arts education, primarily music and visual art, as one of the key areas of focus in pursuing the goal of holistic education of a primary school child in the Singapore school system.	The survey research was carried out in music education involving musical learnings from early childhood through tertiary education with a view to identify key areas of research interests and gaps.
	del Barrio *et al.* (2009)	The authors use a multivariate statistical technique to synthesise the initial information and data envelopment analysis (DEA) for efficiency evaluation in regional heritage institutions in Spain.	The results point to the promotion of evaluation tools and methods for management of heritage institutions and public resource allocation.
	Schuele & Lederman, 2004	This study aims to explore common work-related injuries among instrumental musicians, assessing the risk for long-term disability.	The authors found that many of the medical problems encountered in instrumental musicians seem to have a good outcome and rarely lead to long-term disability except for focal dystonia. Work-related disorders might be eligible for health and wage-loss benefits through workers’ compensation and private and state-funded disability insurance.
	Gibson, 2003	This article explores students’ and teachers’ attitudes towards art and arts education.	The author found that the prior experiences, existing knowledge, beliefs, attitudes, perceptions, and interest in the visual arts of student teachers impacts the likelihood of their adding visual arts education to teaching curricula in a primary school context.
**Health/** **well-being** **(2 articles)**	Conquergood, 1988	This article studies the effects of an environmental health education program, which employed performances and popular theatre, on critical awareness about the health problems: 1) among refugees in Ban Vinai, dealing with trauma and crisis; and 2) implications for medical health officials.	The author argues that for health care programs to be delivered successfully, agency workers depend on the acceptance and cooperation of the recipients. A result of the study pointed to the need for more consciousness-raising activities for health professionals themselves. “For popular theatre to work effectively as a tool of critical awareness and empowerment for oppressed peoples it must be rooted in and begin with their cultural strengths” (p. 181).
	Edmondson, 2005	The study explores hoe theatre and performance can be used to market trauma and humanitarianism with the case of refugees in northern Uganda.	The author is critical to how arts therapy in northern Uganda was valued primarily to market trauma. “These works did not function as personal expressions of trauma and healing; instead, they were assimilated into the master narrative of war.” (p. 457). “In the creation of the World Vision plays, the staging methods worked to silence the children's voices, as their own ideas about children's rights or peacemaking were never solicited” (p. 461).
**Community** **empowerment/identity** **(1 article)**	Sonn *et al.*, 2015	This article explores the potential in photography and photo elicitation as a medium for self-expression and place “among aboriginal and non-indigenous children, young people, and adults in four rural towns in Australia” (p. 89).	(The method) “reflected individual and collective constructions of place, based on positive experiences and emotions tied to the natural environment and features of the built environment (...) “it is an approach that can contribute to community psychology’s empowerment agenda” (ibid).
**Theory/method** **development** **(8 articles)**	Gregory, 2003	Study of assessment tool’s ability to capture a scientific theory’s popularisation via popular press, museums, etc.	The paper argues for science communication studies to look beyond traditional categories to embrace the wide variety of media and genres that contribute to the construction of science in the public.
	Vannini & Waskul, 2006	In this article, the authors argue for the creation of the metaphor life as music.	From within the metaphor of life as music, the authors conceptualize beauty as the diverse rhythms, melodies, and harmonies contributing to the constitution of both subjectivity and intersubjectivity.
	Miller, 2008	This article investigates the *Grand Theft* Auto video game series in order to demonstrate the potential of a folkloristic, ethnographic approach for the analysis of digital games.	This case study suggests that digital gameplay should be regarded as a form of performance practice with the capacity to invoke traditional folkloric genres and engender new traditions.
	Campbell, 2008	A discussion of artistic projects concerning sharing the past. Theories of memory work, memory sharing as a cultural practise.	Shared experiences “allows us to forge a usable past together.”
	Neelands, 2009	Discussing the ensemble-based approach of drama in schools. Contrasts the pro-social emphasis in the ensemble model with a pro-technical emphasis.	Using ideas drawn from McGrath and Castoriadis, the paper claims that the ensemble approach provides young people with a model of democratic living.
	Hughes, 2012	Effects of cultural interpretations of a phenomenon (nuclear culture) on popular imagery.	Risk of homogeneous storytelling, reduction of pluralistic histories.
	Horton & Berlo, 2013	An analysis of why it has been difficult for the ‘new materialisms’ to incorporate indigenous intellectual traditions into discussions of non-human agency, focusing on contemporary arts discourse.	Indigenous artists’ understandings of material have an acute awareness of the contemporary, global challenges of cohabitation.
	Taylor, 2013	This article analyses how affective listening is used to develop performances in Alecky Blythe's verbatim theatre.	The article argues that voice in *London Road* both claims and defers authenticity and authority, since voice signifies presence and embodied identity but the reworking of speech into song signals the absence of the real.
**Skills enhancement** **(7 articles)**	Lindstrom, 2006	Can the arts be assessed and taught? A study of portfolios as methods for art skills enhancement among young people.	The notion that assessments of learning outcomes must be either limited to superficial knowledge or completely arbitrary is shown to be a misconception. Improved visual design and artistic skills can be done in certain circumstances.
	Moreno *et al.*, 2011	The effect of 20 days of music or visual arts training on children’s pre-literacy skills.	(The children’s) “ability to map unfamiliar symbols to known words improved significantly from pretest to posttest” (p. 170). The effects on the music group were stronger.
	Greenfader *et al.*, 2015.	Effect of a Performing Arts program on the oral language Skills of young English learners	The treatment group ( *N* = 902) outperformed controls ( *N* = 4,338) on speaking assessments. Effects strongest on English learners with the most limited English-speaking abilities.
	Hsiao, 2010	Investigate methods of enhancing kindergarteners' artistic creative thinking and expressive drawing through appreciation of picture books.	Significant and positive change in children's reading and drawing behaviors at home. “The results also showed that the collage series of picture books had more impact on children than did other picture books in terms of teaching efficacy by picture book appreciation.”
	Eerola & Eerola, 2014	Can music education create social benefits in the school environment (general satisfaction about the school and a sense of achievement and opportunity for students)?	Extended music education enhanced the quality of school life and had a positive effect on the social aspects of schooling.
	Rickard *et al.*, 2012.	The impact of an increase in school-based music training on a range of cognitive and psychosocial measures for 10–13-year-olds in two independent studies.	No convincing benefits of school music classes were apparent. “The intrinsic value of music education for enjoyment and learning should therefore remain central to the justification of music education in the national school curriculum” (abstract).
	Moneta & Rousseau, 2008	Emotional regulation (ER) capabilities through drama intervention, immigrant adolescents with behavioral difficulties.	Some impairment in emotional expression and emotional regulation in this study sample. “In general, the drama process seemed to help emotional expression and awareness and to foster a transformation of emotive processes in the sense of a ‘collective ER.’”
**Knowledge** **dissemination/** **learning** **(7 articles)**	Henriksen & Frøyland, 2000	Explore “the potential of museums to provide information and experiences that the audience finds relevant in the context of science-related issues they encounter in their private or civic lives.”	Skeptical attitudes among families and museum professionals towards the role of museums for scientific literacy. No found effects threaten the relevance of museums.
	Ash, 2003	The article studies the effects of museums and museum pedagogy on families' scientific sense-making and learning.	Themes in knowledge arise from both the family members and the museum exhibit.
	Peppler, 2010	Understanding contribution of media arts education in informal settings to learning outcomes among underprivileged youth.	Using new types of software, young people can engage with technology that encourages active learning.
	Tofteng & Husted, 2011	How drama can empower action research processes in the field of unemployment. The article also discusses the reactions of the audience, “to use the plays to show others how life is outside the labor market.” (p. 37).	Theatre-based action research opens a new way to communicate and make visible knowledge and experiences from below that have difficulties reaching the public agenda or influencing structures of power.
	Levy, 2006	Examines the variable representation of Saami prehistory in several Nordic museums.	The presentation of Saami prehistory differs significantly between majority community museums and those run by Saami communities. ”The national and regional museums diminish or even deny a Saami role in the antiquity of the nation. In contrast, the Saami institutions grant the Saami the same ancientness as the other Nordic populations” (p 143).
	Dawson, 2018	Explore science communication (through popular media and cultural institutions) from the perspective of participants from low-income, minority ethnic backgrounds.	“Social reproduction in science communication constructs a narrow public that reflects the shape, values and practices of dominant groups, at the expense of the marginalised.”
	Leonard, 2010	Studies the effect of museum and museum pedagogy on audiences' sense-making and learning around popular music.	The exhibition “moved beyond the expected to represent a greater diversity of music genres, sounds, performers and dimensions. Value judgements always inform which exhibition ideas are given the green light and shape the way stories are told within displays” (p. 180).
**The impacts of the** **social on art/** **culture** **(7 articles)**	Lauring *et al.*, 2016	Impact of social and monetary contextual information on liking ratings of art.	Paintings with high monetary primes or with high ratings by peers and art experts led to higher participant liking ratings. In contrast, paintings with a low rating by the low-education/income social group led to higher liking ratings by participants. These results provide empirical support for the social “distinction” behavior theory.
	Im, 2008	This essay examines the implication of interculturalism in New Asia and ultimately the relationship between West and East, focusing on Lee Yountaek's production of *Hamlet* that premiered in Seoul in 1996.	Lee Yountaek's Shakespeare reflects the impasse of contemporary Korean society, whose postcolonial reality is obscured by an optimistic idea of interculturalism.
	Balloffet *et al.*, 2014	This article examines the concept of "edutainment" within the museum sector.	Edutainment presents both opportunities and risks: attracting new audiences, particularly young people, but also “Disneyfication” of cultural institutions.
	Janssen *et al.*, 2008	Studies the importance of national context and global hierarchies for arts journalism and the coverage of non-western culture/arts.	International coverage remains concentrated on a few countries, of which the United States has become the most prominent. Although the global diversity of coverage has increased, non- Western countries are still underrepresented.
	Adams, 2017	Examines the cultural change in Uzbekistan through the evolution of European-style theater during the twentieth century.	The article argues that the adoption of this theatrical form was part of a broader project of cultural modernization (...) “an example of a colonial hierarchy of cultures, which deemed European forms to be more advanced than indigenous ones. This orientation makes an investment in indigenous cultural forms less desirable since they are only intelligible on a local level.” (abstract)
	Sapiro, 2015	An empirical study of the circulation of French literature in the United States in the era of globalization.	Upmarket genres like poetry and theatre are better represented than commercial genres. The high centralization of the publishing field in the Francophone area impacts the circulation pattern. A by-product of the stiffening of commercial constraints on the publishing industry, the discourse on the ‘death of French literature’ paradoxically contributes to nourishing the well-founded fiction of national literatures.
	Wheeler, 2003	This article discusses the role of meaning in the institutionalization of performance art between 1970 and 2000.	“The process of institutionalization is shown as a paradox for American avant-garde art” (...) “Thus institutionalization is a process of negotiation shaped by meaning as well as social structure.” (abstract)
**Fostering ethicality** **and moral behavior,** **audiences and** **participants** **(5 articles)**	Bernard, 2014	Discussion of plays thematizing the Israel/Palestine conflict, examining controversies these plays engendered, and the effort to generate empathy and humanitarian feelings, to persuade a viewer to affiliate with a particular struggle or set of beliefs, and to commit herself or himself to action.	(These plays) “remind us that there is no specific politics associated with an empathetic response (...)” Perhaps these plays will persuade new audiences to ask themselves, with Hare, “Are we where we live? Or are we what we think?” (1998, 43, in Bernard, p. 172). Negative media reception.
	Day, 2002	This study investigates the experiences of, and interactions between, participants of a Forum theatre workshop, which addressed the issue of the refugee child at school.	Findings revealed that the workshop was highly relevant to the students, reflecting moral dilemmas which they faced in their everyday lives, as they encountered refugee students at school. This interactive workshop gave them the opportunity to try out moral behaviour, which could potentially be applied to real-life situations.
	Wake, 2013	A study of audience and media reception, and political effects of Australian play about asylum seekers.	Discussion on potential for pain and exploitation. testimonial theatre. “(...) while *Through the Wire* was ethically problematic, it was also politically efficacious” (p. 117). “The play was highly visible in the mainstream media. The realist aesthetic facilitated identification with asylum seekers (ibid.)
	Heddon *et al.*, 2012	A study of effects of one-on-one theatre performance, dialogue and collaboration.	Risk of intervention: the experiential performance proffers hierarchies of experience; invoking the notion of an ‘ideal audience-participant.’ However, also potential to produce more intimate connections, create togetherness in shared experiences.
**Assessment/** **evaluation** **(5 articles)**	Page *et al.*, 2006	Socially orientated contemporary art is making a success in the wider global context, but is omitted as practice in many schools, although the UK government recommends art as part of curriculum.	“Conclusions have been tentatively drawn about how the curriculum may be better served by the use of contemporary art, as well as the means by which new learning methods may be facilitated” (abstract).
	Lum & Dairianathan, 2014	Assess and evaluate the matching of policy and practice, ambition to include arts education, primarily music and visual art, as one of the key areas of focus in pursuing the goal of holistic education of a primary school child in the Singapore school system.	Survey research carried out in music education involving musical learnings from early childhood through tertiary education with a view to identify key areas of research interests and gaps.
	José del barrio *et al.* (2009)	Using a multivariate statistical technique to synthesise the initial information and data envelopment analysis (DEA) for efficiency evaluation in regional heritage institutions in Spain.	Promoting evaluation tools and methods for management of heritage institutions and public resource allocation.
	Schuele & Lederman, 2004	A study of common work-related injuries among instrumental musicians, assessing the risk for long- term disability.	Many of the medical problems encountered in instrumental musicians seem to have a good outcome and rarely lead to long- term disability except for focal dystonia. Work-related disorders might be eligible for health and wage-loss benefits through workers' compensation and private and state-funded disability insurance.
	Gibson, 2003	What do student teachers really think about art and art education?	Student teachers' prior experiences, existing knowledge, beliefs, attitudes, perceptions, and interest in the visual arts impacts the prospects of them adding visual arts education to their teaching in a primary school context.
**Health/** **well-being** **(2 articles)**	Conquergood, 1988	The effects of an environmental health education program, which employed performances and popular theatre, on critical awareness about the health problems: 1. among refugees in Ban Vinai, dealing with trauma and crisis, and 2. implications for medical health officials.	For health care programs to be delivered successfully, agency workers depend on the acceptance and cooperation of the recipients. A result of the study pointed to the need for more consciousness-raising activities for health professionals themselves. “For popular theatre to work effectively as a tool of critical awareness and empowerment for oppressed peoples it must be rooted in and begin with their cultural strengths” (p. 181).
	Edmondson, 2005	The study “explores the ways in which theatre and performance are used to market trauma and humanitarianism in northern Uganda.”	Arts therapy in northern Uganda was valued primarily to market trauma. “These works did not function as personal expressions of trauma and healing; instead, they were assimilated into the master narrative of war.” (p. 457). “In the creation of the World Vision plays, the staging methods worked to silence the children's voices, as their own ideas about children's rights or peacemaking were never solicited. Instead of speaking as commentators on war, they served as the mouthpieces for predetermined messages (p. 461).
**Community** **empowerment/** **identity** **(1 article)**	Sonn *et al.*, 2015	‘Voices’ used photography and photo elicitation as the medium for exploring and expressing a sense of place among aboriginal and non-indigenous children, young people and adults in four rural towns.	(The method) “reflected individual and collective constructions of place, based on positive experiences and emotions tied to the natural environment and features of the built environment” (...) “it is an approach that can contribute to community psychology’s empowerment agenda.” (abstract)

## Mapping the research front: discussion

The results reveal various interpretations of the social impact of the arts that can inform researchers in this field. As we previously demonstrated, the categories overlap. The analysis also found ambiguous, negative, or null results ( Henriksen & Frøyland, 2000; Rickard *et al.*, 2012), which are useful to understand diverse and sometimes ineffective methods for engaging populations with and through the arts.

Arguably, the results demonstrate that the field thematises and investigates a broader topic area than traditional understandings of art. Rather, our corpus includes studies on cultural institutions, education, and popular culture. Also, they seem to reflect individual or group effects rather than effects on a societal level. Few articles in the data discuss an understanding of “the social” in social impact. For example, Henriksen & Frøyland’s (2000) study on the importance of museums for science-related issues for the public is examined through a group of parents who, they argue, represent the wider public. This is not a critique of that conception, but a wish for a deeper understanding of the link between impacts on individuals, groups, and societies.

When authors of articles in our corpus argue that effects are present in their research, and when they claim to have witnessed empathic responses from the audiences of theatre plays, these populations are not surveyed, nor are these effects tested (c.f. Bernard, 2014; Wake, 2013). Rather, these effects are situated as natural outcomes of the structure of the performance itself: “When audiences think about where Rachel stood, until the moment that she stood in front of a bulldozer, they must also think about where they stand themselves” ( Bernard, 2014, p. 170). Our study was not intended to be evaluative, but we sometimes struggled to find evidence for the arguments for these effects (c.f. Daykin *et al.*, 2008). Studies on the effects of theatre participants ( Day, 2002; Heddon *et al.*, 2012) differ from studies of effects on audiences, with a methodology that allows for a discussion of tested results. Daykin *et al.* (2008, p. 261) conclude that it is unlikely that a single method can be found that will serve as a “gold standard” (ibid) in research on the impact of the arts.

The articles aiming towards the theory or method development of their academic field are not obviously relevant to a discussion on the social impact of the arts. Articles analysed as assessments and evaluations of policy geared toward culture and the arts or institutional and educational strategies do not provide much information about the social impact of the arts either, as they take the meaning of art, often as a positive force, as a given. When effects are accounted for, such as in providing young people with a model for a democratic living ( Neelands, 2009) or creating awareness of global challenges ( Horton & Berlo, 2013), these are not claimed but discussed as *possible* outcomes. For example, Schuele & Lederman’s (2004) study of common work-related injuries among instrumental musicians leads to an interesting discussion on the eligibility of musicians to receive wage-loss benefits and state-funded disability insurance, which arguably would (if they were enforced) be a form of social effect. The articles in the categories ‘theory/method development’ and ‘assessment/evaluation’ make up one-third of the total articles, which demonstrates that a large share (two-thirds) of our corpus does not provide any explicit answers to the question of how to identify the social impact of the arts.

The results pointing to a prevalence of research studying the pedagogical effects of various art forms on children in school settings, regarding their academic performance or skills/knowledge gain, allowed us two reflect on two things: first, the limited role of marginalisation issues in research on the social effect of the arts. This leads to our second concern: the appropriateness of including ‘children’ in a definition of marginalised groups. Arguably, children are a relatively powerless group in society, however, far from all children are experiencing marginalisation. In future similar studies, we recommend researchers studying the social impact of the arts to further problematise ‘children’ as a population.

When articles in the research field thematise identifiable social effects as something more comprehensive than the immediate effect of an observed and limited population (children in a school setting), they can be perceived as:

- Reaching the public policy agenda, eliciting media response, or influencing structures of power ( Bernard, 2014; Tofteng & Husted, 2011; Wake, 2013);
- Building indigenous identities as well as establishing the legitimacy of claims to land and heritage ( Levy, 2006);
- Reflecting the shape, values, and practices of dominant groups, at the expense of the marginalised ( Dawson, 2018);
- Obscuring postcolonial realities ( Adams, 2017; Im, 2008);
- Increasing awareness/changing behaviour concerning health problems among populations and practitioners ( Conquergood, 1988); and
- Engaging in community empowerment ( Sonn *et al*., 2015).

There are many points to be made from these results. For example, although Conquergood (1988) conceives of performance art as a tool to create awareness of health issues, the article argues above all for the dependency of interventions on the participant population, and to consider the culturally appropriate means of reaching out to the population on their terms, i.e., the social prerequisites for working with and changing conditions for a group of people through art interventions. Thus, it is reasonable to claim that the article discusses the social effects *on* art rather than the social effects *of* art.

Several other articles reverse the discussion and ask questions: How do new trends in knowledge dissemination affect museums? ( Balloffet *et al*., 2014) What is the importance of national context and global hierarchies for arts journalism covering non-western culture/arts? ( Janssen *et al*., 2008) How are globalising trends towards interculturalism and modernisation mirrored in national/local theatre developments, and with what effects? ( Adams, 2017; Im, 2008) These are a few examples that made us aware that our method requires further refinement to separate studies of the social effects of art from studies on the social effects *on* art.

This brings us to the results of the analysis which point to the articles thematising the negative social effects of the arts. These include marginalising voices and experiences ( Dawson, 2018; Edmondson, 2005; Levy, 2006), evoking negative emotions or ethically problematic assumptions in production and dissemination ( Heddon *et al*., 2012; Moneta & Rousseau, 2008; Wake, 2013), or purposefully obscuring or trivialising colonialism and its atrocities ( Adams, 2017; Im, 2008). These results both confirm and point to a different kind of negative effect than the theories of Bourdieu (1984) and answer the tentative question of Belfiore and Bennett (2007) as regards unquestioned assumptions about art in cultural policy discourse: can the arts be negative? The analysis of our corpus revealed that the arts can indeed have negative effects, depending on how they are used. For example, Tofteng & Husted (2011) claim that “theatre-based action research opens up a new way to communicate and make visible knowledge and experiences from below that have difficulties reaching the public agenda or influencing structures of power” (p. 27). However, when the analysed play reached the audience, “[o]ne of the scenes was met with massive scepticism” and ended in “heated debate” (p. 37). The audience expressed disbelief in the story of the participant about being refused income support. They dismissed the experience as “fantasy or pure fiction” (ibid). The play was subsequently changed to relate a story that the participant never experienced, which undoubtedly must have been a harrowing ordeal for the participant. However, the author does not account for this episode as part of the social effects of art or artistic choices.

In our analysis of the research front, we found no studies that conceptualise the entertainment or enjoyment aspect of arts and culture as a potential effect apart from Rickard, Bambrick & Gill (2012) who emphasise “the intrinsic value of music education for enjoyment and learning” (p.57).

This analysis of the research front adds perspectives but also confirms that the outcome to develop skills or gaining knowledge is the most researched, and validated, type of effect found in the data. In what ways this effect can be claimed to be *social* requires a theoretical discussion on the links between the individual and societal impact of education.

## Conclusion: understanding the social impact of the arts through mapping the research field

Using bibliographic methods, we aimed to define and survey the research area we call the social impact of the arts. We performed this analysis to understand the major themes and characteristics of the field, including what we operationalise as the ‘research front’. As such, we sought to provide insights into categories, patterns, and trends in the field.

Firstly, through identifying relevant databases and search strings, along with using the PIO model for identifying population, intervention, and outcome in the data to retrieve more relevant hits, we created a data set of over 10,000 articles that constituted the research field. Arts and drama education journals were found to be the most related to the field, along with a few journals on practices and theories in museum studies. The number of publications has risen steadily from 2015 onwards, indicating a growing research interest in the social impact of the arts.

Through a co-word analysis, we identified topic clusters within the corpus, consisting of terms often found co-located with each other in the texts. These were categorised as three overlapping but identifiable themes that, through a close reading of the top-cited articles in each theme, were found to constitute distinctive epistemological and methodological subfields. These were categorised as:

- Social sciences/humanities research on the meaning of arts and culture;
- Arts education research on the meaning of arts for learning/skills outcomes; and
- Research on art as a means of health and well-being.

Through the analysis of these themes, we can understand the social impact of the arts as relating to health and well-being, education, knowledge (or cognitive learning skills), community, and identity. Where the PIO categories are distinguishable, the most studied population was children in a formal learning context. Theatre and drama are the most common interventions, and knowledge/skills enhancement is the most common outcome. This reflects the dominance of arts education journal articles in the corpus.

We conceptualised the research front as a collection of the most cited articles in our corpus. Analysing these articles involved using the PIO model to distinguish research categories and discuss the meaning of the social impact of the arts as described in the data. We concluded that the concept of ‘art’, defined as aesthetical activities, is too narrow to understand the kinds of cultural and artistic interventions and themes explored in the data and needs to be reframed to include a more general ‘culture’ category that can encompass the actions of institutions. The research front of the social impact of the arts investigates populations experiencing marginalisation, such as minorities, refugees, and other disadvantaged groups. However, we also encountered articles relating to a more general media/popular culture theme, which illustrated a desire present in many of the articles to develop theoretical and methodological frameworks. This theme, along with articles aiming to evaluate/assess cultural policy or cultural management tools, added little to the definition of the social impact of the arts, as they either take the social good of the arts for granted, or aim to add to their research field without theorising the significance of their results beyond discipline-specific theoretical and methodologic development. More close reading of the articles – with clearly defined exclusion criteria – is needed to further investigate the meaning of the social impact of the arts in research.

Our analysis also revealed a strong theme in the corpus of fostering ethical and moral behaviour in audiences and participants, health/wellbeing, community empowerment/identity, and most commonly, skills enhancement and knowledge dissemination/learning. Although this study did not have an evaluative aim, it was sometimes difficult to understand if and how the impact has occurred, especially regarding fostering ethical and moral behaviour among audiences. Another theme we discussed was the investigation of how various social issues impact art and artistic practices, which gives an indication that impact is not one-sided. Above we outlined the negative impacts of the arts that we found in our corpus, with examples such as using the arts to silence marginalised voices, to evoke negative emotions or ethically problematic assumptions in production and dissemination, and to obscure or make light of colonial pasts in artistic production.

In the article, we have discussed the meaning of the social effects of the arts. Previous research on the topic often details the impact of the arts on groups or individuals, but seldom theorises links between individual, group, and societal impact. Beyond the enhancement of knowledge and skills as a result of various art interventions, the effects outlined in the research that can be claimed to be social and ‘positive’ are: reaching the public policy agenda; eliciting media response (which can be negative); influencing structures of power; building or shoring up indigenous or community identities as a way of resisting majority cultures; and achieving critical awareness and changed behaviour concerning health problems (in specific circumstances). Few articles in our corpus explored the enjoyment or entertainment aspect of arts and culture. Through the methodology we employed to construct the research front, we developed categories to better understand how the social impact of the arts is studied in research. Future studies could dive deeper into understanding and elaborating on our categories. It would also be useful to perform an expanded close reading from our corpus to either challenge, provide nuance for, or confirm our results.

## Data availability

All data underlying the results are available as part of the article and no additional source data are required.

## Footnotes

^1^The model in nursing research is called PICO (population-intervention-comparison-outcome). As the research on the impact of the arts is more likely to be performed in the social sciences and humanities rather than the natural sciences and medicine with their different epistemological foundations, the category “comparison” was deemed to have lesser importance in our study.
