# Supplementary material for: Mapping research on the social impact of the arts: what characterises the field?
Source: Open Res Eur. 2022 Aug 18;1:124. Originally published 2021 Oct 18. [Version 2] doi: 10.12688/openreseurope.14147.2 (PMC10445867; doi:10.12688/openreseurope.14147.2)
Supplement: Peer review history

# Peer review: Reviewer response for version 2

**DOI:** 10.21956/openreseurope.16148.r29926

**Published:** 2022-09-15

**Authors:** Jennifer Novak-Leonard

**Affiliations:** 1 University of Illinois at Urbana-Champaign, Champaign, IL, USA

No further comments.

If any, are all the source data and materials underlying the results available?

Yes

Is the work original in terms of material and argument?

Yes

Is the work clearly and cogently presented?

Yes

Is the argument persuasive and supported by evidence?

Yes

Does the research article contribute to the cultural, historical, social understanding of the field?

Yes

Does it sufficiently engage with relevant methodologies and secondary literature on the topic?

Yes

Reviewer Expertise:

cultural policy, mixed methods, social impact of arts

I confirm that I have read this submission and believe that I have an appropriate level of expertise to confirm that it is of an acceptable scientific standard.

# Peer review: Reviewer response for version 2

**DOI:** 10.21956/openreseurope.16148.r29927

**Published:** 2022-09-01

**Authors:** Nanna Kann-Rasmussen

**Affiliations:** 1 University of Copenhagen, Copenhagen, 2300, Denmark

The authors have responded in a satisfactory manner and the article has improved.

If any, are all the source data and materials underlying the results available?

Yes

Is the work original in terms of material and argument?

Yes

Is the work clearly and cogently presented?

Yes

Is the argument persuasive and supported by evidence?

Yes

Does the research article contribute to the cultural, historical, social understanding of the field?

Yes

Does it sufficiently engage with relevant methodologies and secondary literature on the topic?

Yes

Reviewer Expertise:

Cultural Policy, Information studies

I confirm that I have read this submission and believe that I have an appropriate level of expertise to confirm that it is of an acceptable scientific standard.

# Peer review: Reviewer response for version 1

**DOI:** 10.21956/openreseurope.15252.r27842

**Published:** 2021-12-20

**Authors:** Jennifer Novak-Leonard

**Affiliations:** 1 University of Illinois at Urbana-Champaign, Champaign, IL, USA

This article aims to contribute to literature on the “social impacts of the arts.” By applying bibliometric methods to relevant “peer-reviewed research in English” (p3), the article seeks to describe trends and the general nature of the “broad and undefined” (p1) field of study on the social impacts of the arts.

 Overall, the article provides a novel approach to describing what comprises the field of research on social impacts of the arts and to “illustrat[ing] the potential variety in the totality of different understandings of art’s social impact displayed in the research literature” (p3). The article is well organized and well written, providing reasoning for the bibliometric methods utilized and generally sufficient specificity to explain choices made in the process of implementing the bibliometric analyses.

 In reading this article, several matters arise for further consideration:

- The article states that its goals are to “inform debates on the social effects of the arts and provide cultural policy scholars and artist-researchers with insights into categories, patterns, and trends in the research field of the social impact of the arts” (p3); while the latter is well addressed throughout the article, further reflection on the former and the implications of the article’s findings about the state of research for debates in spheres of policy and/or practice could strengthen the article.     
      
- Given the article’s definition of ‘“social impact” as relating to issues of marginalization” (p3) and finding that “Marginalisation issues, such as minorities and refugees, are not the most prominent themes in terms of populations in research” (p12), further reflection on why this may be the case or what this suggests for debates on social effects of the arts would be valuable.
- The “dominance of arts education” (p21) throughout the bibliometric analyses, as well as health and therapy, raises questions about how the more advanced maturity of these fields of study is being considered in terms of the operationalized definition of “social impacts of the arts” used within this article. These more mature fields of study seem to align themselves more readily with the PIO framework, the search terms chosen (Table 1), and – particularly in the case of arts education – publishing using certain conventions and terminology developed by the respective field, as seen in Table 3. Further reflection on less mature fields or areas of study, or further reflection on limitations of the bibliometric analyses to address more emergent, relevant areas of study would be valuable.

In sum, the article makes a notable contribution to literature on the “social impacts of the arts” and offers a novel bibliometric analytical approach to exploring the state of the research literature. Further critical reflection on relevant considerations in context of the policy debates motivating this study could help readers better understand the importance and implications of this article.

If any, are all the source data and materials underlying the results available?

Yes

Is the work original in terms of material and argument?

Yes

Is the work clearly and cogently presented?

Yes

Is the argument persuasive and supported by evidence?

Yes

Does the research article contribute to the cultural, historical, social understanding of the field?

Yes

Does it sufficiently engage with relevant methodologies and secondary literature on the topic?

Yes

Reviewer Expertise:

cultural policy, mixed methods, social impact of arts

I confirm that I have read this submission and believe that I have an appropriate level of expertise to confirm that it is of an acceptable scientific standard, however I have significant reservations, as outlined above.

# Response

**Published:** 2022-06-28

**Authors:** Sofia Lindström Sol

**Affiliations:** University of Borås, Sweden

We, the authors, would like to thank the reviewers for their time and effort in bringing some issues in our paper to light. We are indebted to their thorough yet supportive comments, which have improved the article. Below, I will summarize each reviewer's comments and add a reply with how we have tried to address their concerns in the article.

***R2. 1*** *The article states that its goals are to "inform debates on the social effects of the arts and provide cultural policy scholars and artist-researchers with insights into categories, patterns, and trends in the research field of the social impact of the arts" (p3); while the latter is well addressed throughout the article, further reflection on the former and the implications of the article's findings about the state of research for debates in spheres of policy and/or practice could strengthen the article.*

**Reply**: We have revised the introduction and conclusion to better portray what the article actually offers—this means we have slightly downplayed the policy contribution of the article. We do believe that our article can inform policy debates through insights into patterns in research, especially through an understanding of the art's impact as multi-faceted.

***R2. 2** Given the article's definition of '“social impact” as relating to issues of marginalization” (p3) and finding that “Marginalisation issues, such as minorities and refugees, are not the most prominent themes in terms of populations in research” (p12), further reflection on why this may be the case or what this suggests for debates on social effects of the arts would be valuable.*

**Reply:** An important remark. We believe this is a significant result: research on the social impact of the arts does not focus on marginalization issues. This relates to reviewer 1’s third comment about ”normal” school children and how our inclusion of children as a marginalized group impacts our data. We have added a paragraph in the discussion about this, p.15.

   

***R2. 3*** *The “dominance of arts education” (p21) throughout the bibliometric analyses, as well as health and therapy, raises questions about how the more advanced maturity of these fields of study is being considered in terms of the operationalized definition of “social impacts of the arts” used within this article. These more mature fields of study seem to align themselves more readily with the PIO framework, the search terms chosen (Table 1), and – particularly in the case of arts education – publishing using certain conventions and terminology developed by the respective field, as seen in Table 3. Further reflection on less mature fields or areas of study, or further reflection on limitations of the bibliometric analyses to address more emergent, relevant areas of study would be valuable.*

**Reply:** We see more research related to specific fields in the data. Using Web of Science has its limitations—it has the required bibliometric data, but it also comes with a hierarchy of what kinds of research are visible. This method risks a particular bias: the node's size becomes a measurement for relevance, which is why articles from the medical field get a “head-start.” We believe that the next step, suitable for a follow-up article, could examine how different fields are valued, especially concerning policy. Certain effects of the arts may never be measurable—that does not mean they don’t exist. We have added a discussion on these in a few sentences in the methods section, p. 5.

# Peer review: Reviewer response for version 1

**DOI:** 10.21956/openreseurope.15252.r27955

**Published:** 2021-12-08

**Authors:** Nanna Kann-Rasmussen

**Affiliations:** 1 University of Copenhagen, Copenhagen, 2300, Denmark

This article reports from a large-scale review of literature on “the social impact of the arts”.

 The article is not a study of effects, but a bibliometric study aiming to characterize the research field “ *through an overview of trends in studies of the social impact of the arts as a broadly defined research area*” (p.3). The article is driven by a “conceptual ambition” and seeks to analyze the major themes and research fronts in the literature on the social impact of the arts. On p.3 the authors describe an additional objective, “to illustrate the potential variety in the totality of different understandings of art’s social impact displayed in the research literature”.

 Overall, the article is clear and well written. The article has a very good description of the methods used. Even for a non-bibliometric, the article conveys a clear picture of what has been done and how the authors reach their conclusions.

 However, I have a few comments on some issues in the article.

1. The first part of the study is a co-word analysis resulting in three clusters. It is unclear why it is necessary to first describe the three clusters and then compare them. The descriptions, particularly of the “blue” cluster consists only of examples, so I would suggest the authors to shorten the descriptions and elaborate on the “comparison”, even if I don’t think it is a comparison, but just a better and more analytical informed description.
2. The second part of the study regards a mapping of the “research front”. This is done both quantitatively and qualitatively, which works well. However, on pages 10-11 there are quite a few paragraphs regarding methodological considerations of the research front that indicate that the original dataset is not very good. Using bibliographic coupling, the authors identify nine clusters, but when they look at the majority of the clusters, the blue, yellow, orange and brown as well as the purple, turquoise and pink, it turns out that the articles in them are not very relevant after all, and the authors exclude them from their qualitative analysis. This actually leaves only the red and green cluster. As a reader, I missed a reflection on this. If the articles in the seven clusters were not relevant for analysis – why were they kept in the dataset?
3. My last comment regards the concept of social impact. In the section called “Delineating the field” the authors employ a narrow view of social impact (relating solely to “marginalization” e.g. poverty, minority issues, refugees, underserved or marginalised communities). This is not necessarily a problem. However, it seems that in the later parts of the article (especially tables 5, 6, 7) a substantial part of the articles in the dataset are seemingly about “normal” school children that are not necessarily underprivileged or marginalized. The article would benefit from a reflection of this as well. As well as the above comment, this regards the quality of the dataset.

The conclusion as well as the rest of the article is well written and well structured.

If any, are all the source data and materials underlying the results available?

Yes

Is the work original in terms of material and argument?

Yes

Is the work clearly and cogently presented?

Yes

Is the argument persuasive and supported by evidence?

Yes

Does the research article contribute to the cultural, historical, social understanding of the field?

Yes

Does it sufficiently engage with relevant methodologies and secondary literature on the topic?

Yes

Reviewer Expertise:

Cultural Policy, Information studies

I confirm that I have read this submission and believe that I have an appropriate level of expertise to confirm that it is of an acceptable scientific standard, however I have significant reservations, as outlined above.

# Response

**Published:** 2022-06-28

**Authors:** Sofia Lindström Sol

**Affiliations:** University of Borås, Sweden

We, the authors, would like to thank the reviewers for their time and effort in bringing some issues in our paper to light. We are indebted to their thorough yet supportive comments, which have improved the article. Below, I will summarize each reviewer's comments and add a reply with how we have tried to address their concerns in the article.

***Reviewer 1, comment 1:*** *The first part of the study is a co-word analysis resulting in three clusters. It is unclear why it is necessary to first describe the three clusters and then compare them. The descriptions, particularly of the "blue" cluster consist only of examples, so I would suggest the authors to shorten the descriptions and elaborate on the "comparison", even if I don't think it is a comparison, but just a better and more analytical informed description.*

**Reply:** We agree with this comment and have revised the text to focus on comparing the clusters rather than describing each three, hopefully without obfuscating any distinctiveness between them. Examples of articles are still important for the reader, we believe, to illustrate the details in the more generalized analysis of the clusters.

***R1. 2*** *The second part of the study regards a mapping of the "research front". This is done both quantitatively and qualitatively, which works well. However, on pages 10-11 there are quite a few paragraphs regarding methodological considerations of the research front that indicate that the original dataset is not very good. Using bibliographic coupling, the authors identify nine clusters, but when they look at the majority of the clusters, the blue, yellow, orange and brown as well as the purple, turquoise and pink, it turns out that the articles in them are not very relevant after all, and the authors exclude them from their qualitative analysis. This actually leaves only the red and green cluster. As a reader, I missed a reflection on this. If the articles in the seven clusters were not relevant for analysis – why were they kept in the dataset?*

**Reply:** This is a great comment, and we have added a reflection under the headline Surveying a field: a literature review, and under the headline About the clusters. We have also revised this part to reflect better what we did. Our wide selection reflects our keywords: we threw a wide net to see a specific thing and got "junk," which we needed to filter and sort for relevance. We combed wide and focused on the subject clusters that fit our analysis. We see this as a strength of the article! This quantitative approach needs to be supplemented with a qualitative approach, but we did not allow this to guide the analysis. The other themes are not irrelevant to the topic but to our specific aim of exploring social impact concerning marginalization as a broad concept.

***R1.3*** *My last comment regards the concept of social impact. In the section called "Delineating the field" the authors employ a narrow view of social impact (relating solely to "marginalization" e.g. poverty, minority issues, refugees, underserved or marginalised communities). This is not necessarily a problem. However, it seems that in the later parts of the article (especially tables 5, 6, 7) a substantial part of the articles in the dataset are seemingly about "normal" school children that are not necessarily underprivileged or marginalized. The article would benefit from a reflection of this as well. As well as the above comment, this regards the quality of the dataset.*

**Reply:** We agree and have added a reflection about this in the discussion section (p.15). Using 'children' as a marginalized group was part of the research project and drove the aim at hand, but the results tell us that using 'children' as a marginalized group in searches needs to be done with caution in future studies.
